# Mortality for Time-Sensitive Conditions at Urban vs Rural Hospitals During the COVID-19 Pandemic

**DOI:** 10.1001/jamanetworkopen.2024.1838

**Published:** 2024-03-12

**Authors:** H. Joanna Jiang, Rachel M. Henke, Kathryn R. Fingar, Lan Liang, Denis Agniel

**Affiliations:** 1 Agency for Healthcare Research and Quality, Rockville, Maryland; 2 Now with Lewin Group, Boston, Massachusetts; 3 IBM Watson Health, Santa Barbara, California; 4 Now with Everytown for Gun Safety, New York, New York; 5 RAND Corporation, Santa Monica, California

## Abstract

**Importance:**

COVID-19 pandemic-related disruptions to the health care system may have resulted in increased mortality for patients with time-sensitive conditions.

**Objective:**

To examine whether in-hospital mortality in hospitalizations not related to COVID-19 (non–COVID-19 stays) for time-sensitive conditions changed during the pandemic and how it varied by hospital urban vs rural location.

**Design, Setting, and Participants:**

This cohort study was an interrupted time-series analysis to assess in-hospital mortality during the COVID-19 pandemic (March 8, 2020, to December 31, 2021) compared with the prepandemic period (January 1, 2017, to March 7, 2020) overall, by month, and by community COVID-19 transmission level for adult discharges from 3813 US hospitals in the State Inpatient Databases for the Healthcare Cost and Utilization Project.

**Exposure:**

The COVID-19 pandemic.

**Main Outcomes and Measures:**

The main outcome measure was in-hospital mortality among non–COVID-19 stays for 6 time-sensitive medical conditions: acute myocardial infarction, hip fracture, gastrointestinal hemorrhage, pneumonia, sepsis, and stroke. Entropy weights were used to align patient characteristics in the 2 time periods by age, sex, and comorbidities.

**Results:**

There were 18 601 925 hospitalizations; 50.3% of patients were male, 38.5% were aged 18 to 64 years, 45.0% were aged 65 to 84 years, and 16.4% were 85 years or older for the selected time-sensitive medical conditions from 2017 through 2021. The odds of in-hospital mortality for sepsis increased 27% from the prepandemic to the pandemic periods at urban hospitals (odds ratio [OR], 1.27; 95% CI, 1.25-1.29) and 35% at rural hospitals (OR, 1.35; 95% CI, 1.30-1.40). In-hospital mortality for pneumonia had similar increases at urban (OR, 1.48; 95% CI, 1.42-1.54) and rural (OR, 1.46; 95% CI, 1.36-1.57) hospitals. Increases in mortality for these 2 conditions showed a dose-response association with the community COVID-19 level (low vs high COVID-19 burden) for both rural (sepsis: 22% vs 54%; pneumonia: 30% vs 66%) and urban (sepsis: 16% vs 28%; pneumonia: 34% vs 61%) hospitals. The odds of mortality for acute myocardial infarction increased 9% (OR, 1.09; 95% CI, 1.06-1.12) at urban hospitals and was responsive to the community COVID-19 level. There were significant increases in mortality for hip fracture at rural hospitals (OR, 1.32; 95% CI, 1.14-1.53) and for gastrointestinal hemorrhage at urban hospitals (OR, 1.15; 95% CI, 1.09-1.21). No significant change was found in mortality for stroke overall.

**Conclusions and Relevance:**

In this cohort study, in-hospital mortality for time-sensitive conditions increased during the COVID-19 pandemic. Mobilizing strategies tailored to the different needs of urban and rural hospitals may help reduce the likelihood of excess deaths during future public health crises.

## Key Points

**Question:**

How did patient outcomes for non–COVID-19 time-sensitive conditions change during the pandemic?

**Findings:**

In this cohort study of 3813 US hospitals, including 18 601 925 hospitalizations, odds of in-hospital mortality increased significantly for non–COVID-19 sepsis and pneumonia during the pandemic (March 8, 2020, to December 31, 2021) compared with before the pandemic (January 1, 2017, to March 7, 2020) at both urban and rural hospitals, but the increase was greater at rural hospitals. In-hospital mortality significantly increased for acute myocardial infarction and gastrointestinal bleeding at urban hospitals and for hip fracture at rural hospitals.

**Meaning:**

In this study, patient outcomes for time-sensitive conditions were worse during the pandemic than before, with different magnitudes of change at urban vs rural hospitals, suggesting that strategies tailored to the different needs of these hospitals may reduce mortality during future public health crises.

## Teaser

This cohort study assesses in-hospital mortality among adults at US rural and urban hospitals during vs before the COVID-19 pandemic for non–COVID-19 time-sensitive conditions.

## Introduction

All-cause inpatient mortality rates have soared since the onset of the COVID-19 pandemic.^1,2^ An obvious reason for this increase is the high risk of mortality among COVID-19 hospitalizations, particularly during peaks of the pandemic.^3^ However, increased mortality for non–COVID-19 hospitalizations has also been documented in studies from multiple countries.^4,5,6,7,8,9^ Possible explanations include patients’ delay in seeking care out of fear for COVID-19 infection at hospitals^10^; postponement of elective surgery to accommodate COVID-19 surges,^11^ which in some cases could affect patients’ health; strain of hospital resources such as staffing, intensive care unit beds, and medical supplies for caring for patients with COVID-19^12^; and other factors such as staff burnout and strict visitor limitations that may compromise patient care.^13^

The spillover impact of the pandemic on patients without COVID-19 may be particularly concerning for time-sensitive conditions such as acute myocardial infarction (AMI) or sepsis, in which delayed care due to patient and/or system factors could lead to more severe cases and worse outcomes. In this study, we focused on hospitalizations for 6 conditions widely regarded as time sensitive: AMI, hip fracture, gastrointestinal (GI) hemorrhage, pneumonia, sepsis, and stroke. Although some cases of pneumonia and sepsis may be related to COVID-19, these 2 conditions have a high mortality risk even among patients without COVID-19 and thus should have been monitored during the pandemic. Moreover, sepsis had been increasing prior to the pandemic^14^ and became the most common principal diagnosis among nonmaternal hospitalizations in the US.^15^

We leveraged all-payer hospital discharge data from 45 states and the District of Columbia, which provided a more representative patient sample than those found in prior studies using either single-payer data (eg, Medicare) or smaller samples. The objective of our study was to quantify excess hospital deaths in patients hospitalized for non–COVID-19 conditions (non–COVID-19 stays) during the pandemic (from March 8, 2020, to December 31, 2021) compared with the prepandemic period (from January 1, 2017, to March 7, 2020), overall, by month, and by level of community COVID-19 transmission, at urban and rural hospitals. Delayed care and resource strain may have increased at times of high COVID-19 transmission in the community. Furthermore, given differences in population density, magnitude and timing of the pandemic, and availability of health care resources between urban and rural areas, it is important to examine whether the pandemic’s spillover impact for non–COVID-19 conditions may be different at urban vs rural hospitals.

## Methods

### Data Sources and Study Population

This cohort study used data from the State Inpatient Databases for the Healthcare Cost and Utilization Project of the Agency for Healthcare Research and Quality.^16^ We included inpatient stays from 3813 community general acute care hospitals in 45 states and the District of Columbia for 2017 through 2021 (see eTable 1 in Supplement 1). We used the Clinical Classifications Software Refined^17^ for the *International Classification of Diseases, Tenth Revision, Clinical Modification* (*ICD-10-CM*) to identify COVID-19 diagnoses and to define stays for time-sensitive conditions with a principal diagnosis of AMI, hip fracture, GI hemorrhage, pneumonia, sepsis, and stroke (eTable 2 in Supplement 1). We screened all listed diagnoses (the principal diagnosis plus secondary conditions) to identify COVID-19. For each time-sensitive condition, we separated stays with vs without a COVID-19 diagnosis. We excluded patients who were transferred to another acute care hospital because the patient was no longer at risk of death in the original hospital. This study followed the Strengthening the Reporting of Observational Studies in Epidemiology (STROBE) reporting guideline for cohort studies.^18^ This study was determined by the Agency for Healthcare Research and Quality to be exempt from Institutional Review Board review as it does not constitute research involving human subjects defined under Code of Federal Regulations Title 45 Part 46. 

### Variables

The outcome was in-hospital mortality identified by discharge disposition. The primary independent variable was a discharge (or death) date during the pandemic (after March 7, 2020). The reference group was inpatient stays in the prepandemic period (January 1, 2017, to March 7, 2020). The pandemic period was examined overall and by month and segmented by COVID-19 burden in the hospital’s community. COVID-19 burden was defined by transmission level in the hospital’s county in the 7 days prior to the discharge date. It was categorized by new cases per 100 000 population as low (0 to 9), moderate (10 to 49), substantial (50 to 99), and high (100 or more) according to the Centers for Disease Control and Prevention.^19^

Rural hospitals were defined as those in zip codes eligible for funding by the Federal Office of Rural Health Policy.^20^ Patient characteristics obtained from the Healthcare Cost and Utilization Project included age, sex, race and ethnicity, community income, expected payer, and comorbidities defined by the Elixhauser Comorbidity Software Refined for *ICD-10-CM*.^21^ Race and ethnicity Healthcare Cost and Utilization Project uniform data reflect patient self-report recorded by hospitals and included Asian or Pacific Islander, Black, Hispanic, White, and other (American Indian or Alaska Native and other races).

### Statistical Analysis

In descriptive analyses, we compared patient characteristics of hospital stays for each time-sensitive condition between the prepandemic and the pandemic periods. Due to the large sample size of the State Inpatient Databases, small differences in patient characteristics can be statistically significant. Instead, we used standardized mean differences (SMDs)^22^ to assess balance in patient characteristics between the 2 time periods.

We used entropy weights to align stays in the prepandemic period with stays in the pandemic period with respect to patient age, sex, and Elixhauser comorbidity index score for mortality.^23^ We chose entropy balancing over other matching methods (eg, propensity scores) because it more closely balances the 2 samples and produces less extreme weights while eliminating the need for iteration and postcalibration in probability matching methods.^24,25,26^ It retains all treated patients to produce a more accurate estimation of treatment effects with less computing burden.^27^ In this analysis, we calculated separate entropy weights for each time-sensitive condition, each level of community COVID-19 burden, and each quarter of 2020 and 2021.

We estimated interrupted time-series logistic regression models to assess level shifts in the in-hospital mortality associated with the pandemic compared with the prepandemic period (see the eMethods in Supplement 1 for model specification). We generated separate estimates for rural and urban hospitals. First, we obtained overall changes comparing the pandemic period of 2020 (March 8 to December 31) with the prepandemic period. Second, we estimated separate models for discharges in periods of low, moderate, substantial, and high community COVID-19 burden, each compared with the prepandemic period. Third, we produced month-specific estimates for each month in the pandemic period of March 8, 2020, through 2021, compared with the prepandemic period. Since the 2021 data were available from only a subset of states (eTable 1 in Supplement 1), the monthly estimates were limited to those states contributing data through each quarter of that year. All analyses were performed in Stata, version 17.0 (StataCorp LLC) using a 2-sided *P* = .05 as the threshold for statistical significance.

## Results

### Number of Inpatient Stays and Observed Mortality Rates During the COVID-19 Pandemic

After excluding cases with a diagnosis code of COVID-19, there were 18 601 925 hospitalizations; 49.7% were female and 50.3% male, 38.5% were aged 18 to 64 years, 45.0% were aged 65 to 84 years, and 16.4% were 85 years or older for the selected time-sensitive medical conditions from 2017 through 2021. During the pandemic period of March 8, 2020, through 2021, sepsis was the most common principal diagnosis for hospital stays (Table 1). It had the highest percentage of cases with a secondary diagnosis of COVID-19 (17.6%) compared with the other 5 conditions, among which the percentage of COVID-19 cases ranged from 0.7% to 2.7%. Among these 6 time-sensitive conditions, the observed in-hospital mortality rates for stays with concomitant COVID-19 were higher compared with those for non–COVID-19 stays. The mortality rate was the highest for sepsis for those with COVID-19 compared with those without COVID-19 (25.4% vs 10.0%), followed by AMI (14.0% vs 5.1%), stroke (11.0% vs 3.8%), and pneumonia (9.6% vs 3.4%). The mortality rates were relatively lower for GI hemorrhage (6.9% vs 3.0%) and hip fracture (6.6% vs 1.7%).

**Table 1.** Hospital Stays With and Without a COVID-19 Diagnosis During the Pandemic^a^

Time-sensitive condition^b^	Hospital stays, No. (%)		Observed in-hospital mortality rate, %	
	Patient without COVID-19	Patient with COVID-19	Patient without COVID-19	Patient with COVID-19
Sepsis (n = 2 753 442)	2 267 617 (82.4)	485 825 (17.6)	10.0	25.4
Acute myocardial infarction (n = 637 398)	627 168 (98.4)	10 230 (1.6)	5.1	14.0
Stroke (n = 609 802)	596 560 (97.8)	13 242 (2.2)	3.8	11.0
Pneumonia (n = 521 133)	517 257 (99.3)	3876 (0.7)	3.4	9.6
Hip fracture (n = 378 527)	370 915 (98.0)	7612 (2.0)	1.7	6.6
Gastrointestinal hemorrhage (n = 317 098)	308 652 (97.3)	8446 (2.7)	3.0	6.9
All others (n = 22 585 681)	20 680 230 (91.6)	1 905 451 (8.4)	2.2	10.5

^a^ COVID-19 was identified by screening all listed diagnoses (the principal diagnosis plus secondary conditions); from March 8, 2020, to December 31, 2021.

^b^ Time-sensitive conditions were defined by the principal diagnosis based on *International Classification of Diseases, Tenth Revision, Clinical Modification* codes as reasons for hospital stays (see eTable 2 in Supplement 1).

### Patient Characteristics Before and During the COVID-19 Pandemic

Patient characteristics of non–COVID-19 stays during the pandemic (March 8, 2020, to December 31, 2021) for each of the 6 conditions were comparable with those in the prepandemic period (January 1, 2017, to March 7, 2020) in terms of distribution across age groups, sex, race and ethnicity, community income levels, expected payers, selected comorbidities, and mean comorbidity index score for mortality (Table 2). The absolute values of SMDs were less than 0.10 (full results including SMDs and a complete list of 34 comorbidities are provided in eTable 3 in Supplement 1). These comparisons were performed before entropy balancing.

**Table 2.** Patient Characteristics of Hospital Stays in the Prepandemic Period and of Hospital Stays Without a COVID-19 Diagnosis in the Peripandemic Period by Time-Sensitive Condition^a^

Patient characteristic	Hospitalizations before and during pandemic, %^b^											
	Acute myocardial infarction		Sepsis		Pneumonia		Gastrointestinal hemorrhage		Hip fracture		Stroke	
	Before (n = 1 882 182)	During (n = 627 168)	Before (n = 6 523 717)	During (n = 2 267 617)	Before (n = 1 935 244)	During (n = 517 257)	Before (n = 984 318)	During (n = 308 652)	Before (n = 981 277)	During (n = 370 915)	Before (n = 1 607 018)	During (n = 596 560)
Rural hospital	8.0	8.9	11.2	13.1	21.0	20.8	10.6	11.2	13.5	14.4	8.5	9.0
Urban hospital	92.0	91.1	88.8	86.9	79.0	79.2	89.4	88.8	86.5	85.6	91.5	91.0
Age, y												
18-24	0.1	0.1	2.1	2.0	1.3	1.1	0.7	0.7	0.4	0.5	0.2	0.2
25-44	5.1	5.5	11.7	12.8	8.0	8.2	7.6	7.6	1.8	2.1	4.1	4.3
45-64	37.7	38.5	29.4	29.9	25.8	27.5	27.4	26.9	11.4	11.2	28.9	29.6
65-84	45.6	46.1	42.5	43.1	45.3	47.4	47.2	49.0	48.9	50.6	48.6	49.6
≥85	11.5	9.9	14.3	12.1	19.7	15.8	17.1	15.8	37.6	35.7	18.1	16.2
Sex												
Female	37.7	36.4	50.4	49.2	53.2	50.1	48.0	47.6	67.1	66.0	49.7	49.0
Male	62.3	63.6	49.6	50.8	46.8	49.9	52.0	52.4	32.9	34.0	50.3	51.0
Race and ethnicity												
Asian or Pacific Islander	2.8	3.0	3.1	3.0	2.1	2.0	3.3	3.5	1.8	1.8	3.0	3.1
Black	11.2	10.9	13.0	13.5	12.0	13.4	14.5	14.3	4.5	4.6	17.0	16.9
Hispanic	8.8	8.6	11.0	10.7	8.2	7.5	9.1	8.6	5.7	5.2	8.3	7.9
White	70.7	72.5	67.0	66.5	72.1	71.0	67.7	67.4	83.0	82.9	65.9	65.4
Other^c^	3.6	3.4	3.4	3.1	3.0	2.5	3.1	2.8	2.4	2.2	3.1	2.7
Community income												
Quartile 1 (lowest)	28.6	28.8	29.6	30.2	29.9	31.1	29.1	29.1	23.8	24.0	28.8	28.8
Quartile 2	27.3	27.4	26.5	26.5	27.6	27.7	26.5	26.6	26.5	26.5	26.3	26.4
Quartile 3	23.7	23.6	23.5	23.2	23.1	22.8	23.5	23.6	25.2	25.2	23.8	23.9
Quartile 4 (highest)	18.6	18.7	18.5	18.1	17.7	16.8	19.2	19.0	23.0	22.9	19.5	19.4
Expected payer												
Medicare	57.0	54.9	61.7	59.3	68.4	66.2	66.4	65.6	82.5	81.8	65.2	63.7
Medicaid	9.5	10.1	14.2	15.6	10.6	11.4	10.8	11.3	3.8	4.2	9.3	10.0
Private insurance	25.3	26.1	17.5	17.8	15.8	16.1	16.4	16.3	9.8	9.7	18.8	19.0
Self-pay or no charge^d^	5.2	5.5	4.3	4.7	3.2	3.5	4.1	4.2	1.5	1.6	4.4	4.6
Other	2.8	3.3	2.1	2.5	1.9	2.6	2.1	2.6	2.3	2.6	2.2	2.7
Elixhauser comorbidity index score for mortality, mean^e^	7.00	6.69	12.59	13.15	8.56	9.17	8.7	9.67	5.73	5.84	10.18	10.95
Comorbidity^f^												
Hypertension, complicated	43.5	44.6	33.6	34.8	34.1	37.2	34.3	37.5	27.2	28.9	31.6	34.4
Hypertension, uncomplicated	38.9	38.5	31.6	31.1	35.1	34.1	37.7	36.0	45.7	44.8	54.4	52.7
Heart failure	37.6	38.6	24.4	25.2	26.6	29.0	22.4	24.9	16.0	16.7	16.5	17.6
Chronic pulmonary disease	21.7	20.7	29.5	26.6	44.9	47.1	22.8	23.1	21.8	22.2	15.8	16.3
Diabetes with chronic complications	26.0	27.2	26.1	28.9	19.6	22.0	20.0	22.6	13.6	15.5	23.3	25.7
Diabetes without chronic complications	14.7	13.8	11.4	10.3	12.4	11.7	12.3	10.7	10.0	9.1	16.0	14.6
Deficiency anemias	15.3	15.1	27.7	29.4	23.7	25.9	24.5	26.4	17.9	19.0	11.4	12.5
Kidney failure, moderate	15.2	15.2	15.5	16.1	13.8	14.8	15.5	17.2	14.1	15.6	12.9	14.0
Kidney failure, severe	7.7	7.2	8.3	8.5	8.1	8.4	9.7	10.5	4.5	4.6	4.1	4.3
Obesity	20.4	23.7	18.7	22.0	15.2	18.7	14.0	16.6	6.1	7.3	14.4	17.1
Depression	9.2	9.8	13.4	13.6	14.4	15.6	13.1	13.7	15.4	16.2	11.3	11.8

^a^ COVID-19 prepandemic time period: January 1, 2017, to March 7, 2020; COVID-19 peripandemic time period: March 8, 2020, to December 31, 2021.

^b^ Differences in each characteristic between the 2 time periods were assessed using standardized mean differences (SMDs). The absolute values of SMDs were less than 0.10 (well below 0.15), indicating similar patient characteristics across the 2 time periods (eTable 3 in Supplement 1).

^c^ Other race and ethnicity includes American Indian or Alaska Native and other races.

^d^ Self-pay or no charge includes self-pay, no charge, charity, and no expected payment.

^e^ Higher scores indicate greater risk of mortality based on the presence of the comorbidities.

^f^ See eTable 3 in Supplement 1 for a complete list of 34 comorbidities.

### Overall Changes in Non–COVID-19 In-Hospital Mortality During the Pandemic

Table 3 presents excess mortality after applying condition-specific entropy weights to adjust for differences in patient age, sex, and comorbidity index score for mortality. Compared with the prepandemic period, odds of in-hospital mortality among non–COVID-19 stays increased during the pandemic at both rural and urban hospitals for pneumonia (rural: odds ratio [OR], 1.46; 95% CI, 1.36-1.57; urban: OR, 1.48; 95% CI, 1.42-1.54) and for sepsis (rural: OR, 1.35; 95% CI, 1.30-1.40; urban: OR, 1.27; 95% CI, 1.25-1.29). Noteworthy, the increase in the odds of mortality for sepsis was greater for stays at rural hospitals than at urban hospitals (35% vs 27%; *P* = .002). Increased odds of mortality during the pandemic were found only at urban hospitals for AMI (9%; OR, 1.09; 95% CI, 1.06-1.12) and for GI hemorrhage (15%; OR, 1.15; 95% CI, 1.09-1.21). For hip fracture, only stays at rural hospitals had increased odds of mortality (OR, 1.32; 95% CI, 1.14-1.53). There was no change in the odds of mortality among stays for stroke during the pandemic compared with the prepandemic period.

**Table 3.** Odds of In-Hospital Mortality in Rural vs Urban Hospitals by Time-Sensitive Condition During the COVID-19 Pandemic Compared With the Prepandemic Period^a^

Time-sensitive condition without a COVID-19 diagnosis^b^	Odds ratio (95% CI)	
	Rural	Urban
Acute myocardial infarction	1.06 (0.98-1.16)	1.09 (1.06-1.12)^c^
Gastrointestinal hemorrhage	1.08 (0.94-1.26)	1.15 (1.09-1.21)^c^
Hip fracture	1.32 (1.14-1.53)^c^	1.05 (0.99-1.11)
Pneumonia	1.46 (1.36-1.57)^c^	1.48 (1.42-1.54)^c^
Sepsis	1.35 (1.30-1.40)^c^	1.27 (1.25-1.29)^c^
Stroke	1.05 (0.95-1.17)	1.00 (0.97-1.03)

^a^ COVID-19 prepandemic time period: January 1, 2017, to March 7, 2020; COVID-19 peripandemic time period: March 8, 2020, to December 31, 2020 (because not all states had data available in 2021). Data were controlled for age, sex, and comorbidity.

^b^ Time-sensitive conditions were defined by the principal diagnosis based on *International Classification of Diseases, Tenth Revision, Clinical Modification* codes as reasons for hospital stays (see eTable 2 in Supplement 1); COVID-19 was identified by screening all listed diagnoses (the principal diagnosis plus secondary conditions).

^c^ Statistically significant at *P* < .001.

### Sensitivity of Excess In-Hospital Mortality to Community COVID-19 Levels

**Figure 1.**
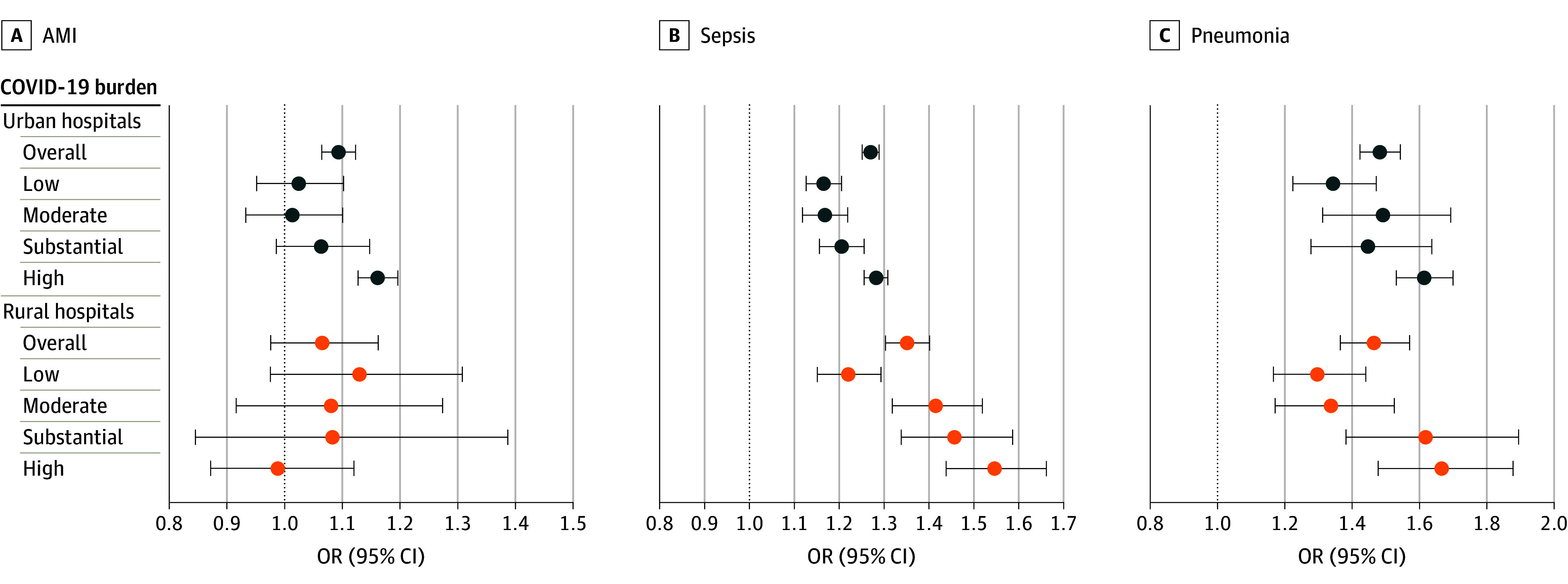
Odds of In-Hospital Mortality Among Non–COVID-19 Stays for Acute Myocardial Infarction (AMI), Sepsis, and Pneumonia During the Pandemic, Overall and by COVID-19 Burden in the Hospital’s Community Compared With Prepandemic Stays Because not all states had data available in 2021, the results are limited to the 2020 peripandemic period (March 8 to December 31, 2020). OR indicates odds ratio.

Compared with the prepandemic period, the odds of in-hospital mortality among non–COVID-19 stays for pneumonia and sepsis were elevated with community COVID-19 burden (low vs high) for a dose-response association (Figure 1 and eTable 4 in Supplement 1). Excess in-hospital mortality during the pandemic at both rural and urban hospitals was the smallest during periods of low community COVID-19 burden but was the largest during periods of high burden. For instance, compared with the prepandemic period, the odds of mortality among stays for sepsis at rural hospitals were 22% higher (OR, 1.22; 95% CI, 1.15-1.29) during periods of low COVID-19 burden and 54% higher (OR, 1.54; 95% CI, 1.44-1.66) during periods of high COVID-19 burden and were 30% higher (OR, 1.30; 95% CI, 1.17-1.44) and 66% higher (OR, 1.66; 95% CI, 1.48-1.88) for pneumonia. A similar pattern was observed for sepsis stays at urban hospitals, with the smallest increase in odds of mortality (16% higher) during low COVID-19 burden periods (OR, 1.16; 95% CI, 1.13-1.20) compared with the largest increase (28% higher) during high burden periods (OR, 1.28; 95% CI, 1.25-1.31) and 34% higher during low COVID-19 burden periods (OR, 1.34; 95% CI, 1.22-1.47) compared with 61% higher during high burden periods (OR, 1.61; 95% CI, 1.53-1.70) for pneumonia.

Among non–COVID-19 stays for AMI, this pattern was observed only at urban hospitals. Excess in-hospital mortality compared with the prepandemic period was higher when the community COVID-19 burden was high (OR, 1.16; 95% CI, 1.13-1.20) or substantial (OR, 1.06; 95% CI, 0.99-1.15). There were no mortality shifts for AMI stays at rural hospitals in response to different COVID-19 burdens. For GI hemorrhage and hip fracture, the odds of mortality during the pandemic compared with the prepandemic period was not associated with a dose response with the community COVID-19 burden (eFigure 1 in Supplement 1). Finally, there was no change in the odds of mortality among stays for stroke associated with the community COVID-19 burden.

### Monthly Variation in Excess In-Hospital Mortality

**Figure 2.**
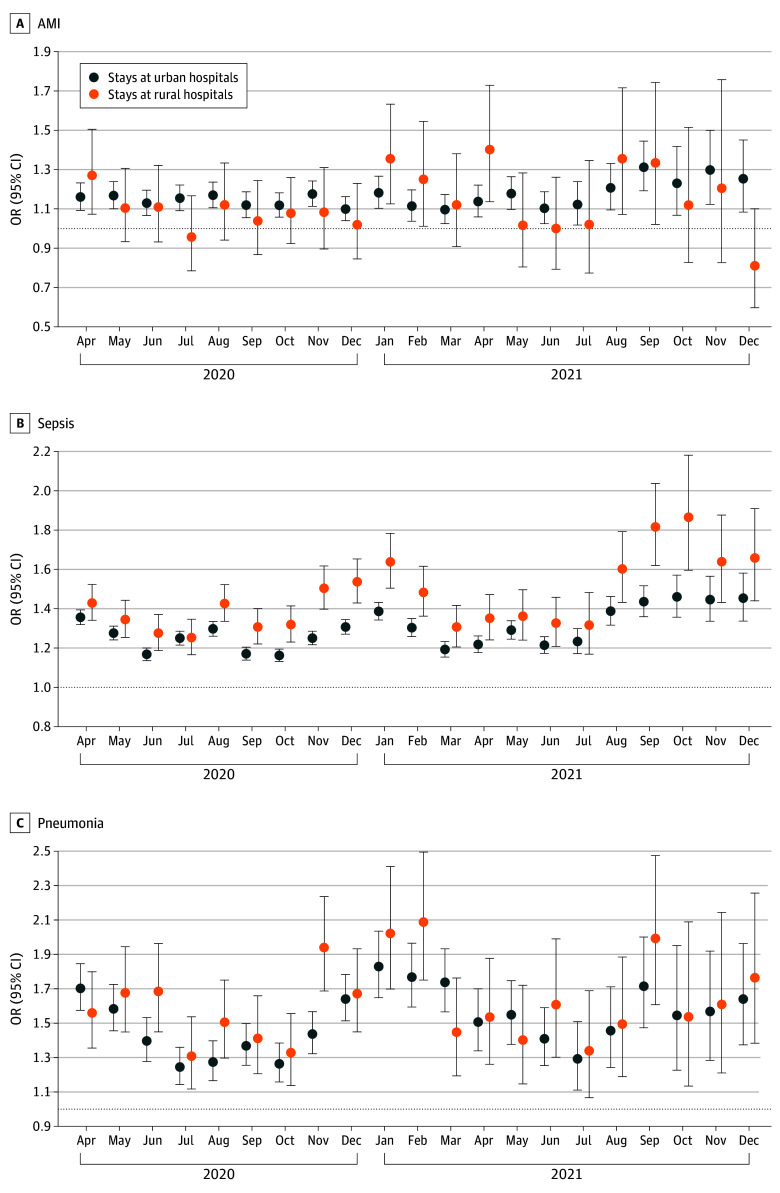
Odds of In-Hospital Mortality Among Non–COVID-19 Stays for Acute Myocardial Infarction (AMI), Sepsis, and Pneumonia in 2020 and 2021, by Month, Compared With Prepandemic Stays From April to December 2020, the data included 45 states and the District of Columbia; January to March 2021, 27 states; April to June 2021, 26 states; July to September 2021, 20 states; and October to December 2021, 12 states. OR indicates odds ratio.

Compared with the prepandemic period, the odds of in-hospital mortality for non–COVID-19 stays for sepsis increased at both rural and urban hospitals in each month of the pandemic through the end of 2021 (Figure 2 and eTable 5 in Supplement 1). Monthly variation was more dramatic at rural hospitals with higher odds of mortality in the initial complete month of the pandemic (April 2020: OR, 1.43; 95% CI, 1.34-1.52), reaching the highest during the winter spanning November and December 2020 to January and February 2021 (eg, January: OR, 1.64%; 95% CI, 1.51-1.79) and another peak in the fall and winter (September through December) of 2021 (eg, October: OR, 1.87; 95% CI, 1.60-2.18). Furthermore, increases in the odds of mortality were significantly greater at rural hospitals than at urban hospitals from August 2020 through February 2021 and again in September and October 2021. The odds of mortality among stays for pneumonia increased in each month of 2020 and 2021 compared with the prepandemic period, displaying similar patterns at both rural (eg, April 2020: OR, 1.56; 95% CI, 1.36-1.80; January 2021: OR, 2.02; 95% CI, 1.70-2.41) and urban (eg, April 2020: OR, 1.70; 95% CI, 1.57-1.85; January 2021: OR, 1.83; 95% CI, 1.65-2.03) hospitals.

However, among stays for AMI, the pattern of monthly variation differed by urban and rural location. Stays for AMI at urban hospitals had increased odds of in-hospital mortality in each month of the pandemic period from April 2020 (OR, 1.16; 95% CI, 1.09-1.23) through December 2021 (OR, 1.25; 95% CI, 1.08-1.45), reaching a peak in September 2021 (OR, 1.31; 95% CI, 1.19-1.45). In contrast, AMI stays at rural hospitals had higher odds of mortality in the initial month of the pandemic in April 2020 (OR, 1.27; 95% CI, 1.07-1.51), the early months of 2021 (eg, January: OR, 1.36; 95% CI, 1.13-1.63), and late summer to early fall of 2021 (eg, August: OR, 1.36; 95% CI, 1.07-1.72).

For the other 3 conditions, excess in-hospital mortality associated with the pandemic occurred only in certain months (eFigure 2 in Supplement 1). Among stays for hip fracture, increased odds of mortality occurred in May 2020 (rural: OR, 1.37; 95% CI, 1.05-1.79; urban: OR, 1.16; 95% CI, 1.02-1.31) and again in late fall (eg, November) of 2020 (rural: OR, 1.43; 95% CI, 1.10-1.87; urban: OR, 1.16; 95% CI, 1.03-1.32) at both rural and urban hospitals. For GI hemorrhage, increased odds of mortality occurred mostly at urban hospitals in specific periods. Increased odds of mortality for stroke occurred at rural and urban hospitals in certain months but did not always coincide. They tended to occur more frequently at rural hospitals than at urban hospitals and in 2021 more than in 2020.

## Discussion

Using all-payer US hospital discharge data from 45 states and the District of Columbia, this cohort study found an increase in non–COVID-19 inpatient mortality for time-sensitive conditions during the pandemic compared with the prepandemic period, after controlling for patient age, sex, and comorbidities. Increases in inpatient mortality were greater and more persistent (ie, in each month throughout the pandemic) for non–COVID-19 sepsis and pneumonia than for other selected conditions. Increases in the odds of mortality for these 2 conditions were also more sensitive to the community COVID-19 burden. Sepsis and pneumonia could be associated with the virus^28^ and particularly likely to be recorded as suspected COVID-19 given similar clinical features to COVID-19 infection. We excluded in this analysis those stays with concomitant COVID-19, which were found to have substantially higher observed mortality rates than non–COVID-19 stays. Furthermore, we observed a persistently high level of excess mortality for non–COVID-19 sepsis from September through December 2021, during which both COVID-19 hospitalizations and deaths suggested downward trends according to COVID-19 statistics compiled by Johns Hopkins University.^29^ Thus, the increase in hospital mortality for sepsis during the pandemic was unlikely associated with COVID-19 infection.

Findings from our study and other studies consistently suggest an association of the pandemic with increased mortality (in-hospital or 30 days after discharge) for non–COVID-19 sepsis and pneumonia.^4,5,7,8^ Delayed care and resource strain were likely associated with this decline in patient outcomes. Early diagnosis and treatment are critical for improving the survival of patients with sepsis.^30^ Emergency department crowding with patients with COVID-19 could have caused delays in treatment for these patients. For severe sepsis, the need for intensive care and mechanical ventilation may have directly competed with the same resources used for treating patients with severe COVID-19.

Our finding that the odds of inpatient mortality for sepsis suggest a greater increase at rural hospitals than at urban hospitals is particularly concerning. Prior research reported an upward trend in the sepsis-related mortality rate in rural areas from 2010 to 2019, whereas the rate remained flat in urban areas, resulting in a widening gap between rural and urban areas.^31^ Compared with their urban counterparts, rural hospitals operated on slimmer margins with more limited staff and resources prior to the pandemic.^32^ It is not surprising that rural hospitals were more vulnerable to pandemic-related disruptions.^33^ Our finding suggests that the rural-urban disparity in outcomes among patients with sepsis increased during the pandemic.

The results of our study also lend support to concerns about the pandemic’s impact on AMI, another life-threatening condition, for which prior studies have shown mixed results.^4,5,7,8,34,35^ We found increased odds of inpatient mortality for non–COVID-19 AMI at urban hospitals throughout the entire pandemic period of 2020 through 2021 and at rural hospitals in those months corresponding to the initial wave of COVID-19 in 2020 and subsequent waves in 2021. Moreover, there were increases in the odds of mortality at urban hospitals with a dose-response association with the community COVID-19 transmission level. One likely explanation is delayed care as found in multiple studies on AMI hospitalizations and outcomes during the pandemic. Prior research found that patients hospitalized with ST-segment elevation MI (STEMI) during the pandemic were significantly associated with delayed care, resulting in more severe cases and higher risks of in-hospital adverse outcomes.^36^ Fear of COVID-19 infection was the most commonly reported reason for patients with STEMI to avoid hospitalization, followed by confusion with COVID-19 vs STEMI symptoms.^37^ Likewise, reluctance to seek care among patients without STEMI was indicated by a greater reduction in hospitalizations during the COVID-19 lockdown compared with STEMI admissions.^34^ System stress is another factor for worse patient outcomes, as a recent study found lower revascularization rates but higher mortality rates for patients without STEMI who were admitted during periods of high hospital COVID-19 burdens.^35^

For non–COVID-19 GI hemorrhage and hip fracture admissions, changes in odds of in-hospital mortality also varied by urban or rural location. To the extent that increased mortality for GI hemorrhage overall during the pandemic compared with the prepandemic period was found only at urban hospitals but not at rural hospitals, the most likely explanation is patients’ delay in seeking care because of fear of contracting COVID-19. Urban areas experienced much higher levels of COVID-19 transmission than rural communities (eFigure 3 in Supplement 1). On the other hand, increased mortality for hip fracture overall at rural hospitals and during COVID-19 peaks at urban hospitals was more likely associated with resource strain. Prior research found a reduction in the proportion of patients with hip fracture undergoing timely surgery during the COVID-19 outbreak.^38^ Furthermore, given the higher mortality for hip fracture patients at rural vs urban hospitals before the pandemic,^39^ our finding suggests a widening gap between urban and rural areas in hip fracture outcomes during the pandemic.

### Limitations

Our study has several limitations. First, COVID-19 diagnoses may not have been captured on discharge records, elevating observed rates of non–COVID-19 mortality. However, given the financial incentive for hospitals to record COVID-19 diagnoses and the heightened sensitivity among clinicians to COVID-19 symptoms, undercoding of COVID-19 should not have been widespread. Empirical studies validating COVID-19 *ICD-10-CM* codes have found high positive and negative predictive values.^40,41^ Second, given the limitations of administrative data, there may have been unmeasured differences in patient severity. Considering that rural hospitals may have had more difficulty transferring their patients during the pandemic (due to surges of patients with COVID-19 at other hospitals), any unmeasured change in patient severity associated with fewer patients who were sicker (or healthier) being transferred may have led to spurious deterioration (or improvement) in mortality at the rural hospitals. Third, we measured only in-hospital mortality, whereas mortality may have occurred after discharge. If patients were discharged prematurely with a potential risk of postdischarge mortality, our estimates would be conservative. Fourth, COVID-19 tests were lacking during initial months of the pandemic, and the COVID-19 case rate may not have fully measured the community transmission level.

## Conclusions

This cohort study found increases in in-hospital mortality for non–COVID-19 time-sensitive conditions during the pandemic, often with differential magnitudes and temporal patterns at urban and rural hospitals. The pandemic may have intensified urban-rural disparities in treatment and outcomes for some of the time-sensitive conditions. Mobilizing strategies tailored to the different needs of urban and rural hospitals may help reduce the likelihood of excess deaths during future public health crises.

## Notes

Supplement 1. **eMethods.** Model Specification **eTable 1.** Number of States Available by Year and Quarter **eTable 2.** *ICD-10-CM* Study Definitions **eTable 3.** Characteristics of Inpatient Stays in the Prepandemic Period (January 1, 2017–March 7, 2020) and Inpatient Stays Without a COVID-19 Diagnosis in the Peripandemic Period (March 8, 2020–December 31, 2021) **eTable 4.** Full Regression Models Comparing In-Hospital Mortality Among Stays During March 8–December 31, 2020, Overall and by COVID-19 Burden in the Hospital’s Community, Relative to Prepandemic Stays **eTable 5.** Full Regression Models Comparing In-Hospital Mortality Among Non–COVID-19 Stays in 2020 and 2021, by Month, Relative to Prepandemic Stays **eFigure 1.** Odds of In-Hospital Mortality Among Non–COVID-19 Stays for GI Hemorrhage, Hip Fracture, and Stroke During March 8–December 31, 2020, Overall and by COVID-19 Burden in the Hospital’s Community, Relative to Prepandemic Stays **eFigure 2.** Odds of In-Hospital Mortality Among Non–COVID-19 Stays for GI Hemorrhage, Hip Fracture, and Stroke in 2020 and 2021, by Month, Relative to Prepandemic Stays **eFigure 3.** Percentage of Hospitals in Counties With High Community COVID-19 Burden (100+ Cases per 100 000 Population in the Past 7 Days), 2020-2021
eReferences

Supplement 2.
Data Sharing Statement
