# Supplementary material for: Mortality for Time-Sensitive Conditions at Urban vs Rural Hospitals During the COVID-19 Pandemic
Source: JAMA Netw Open. 2024 Mar 12;7(3):e241838. doi: 10.1001/jamanetworkopen.2024.1838 (PMC10933716; doi:10.1001/jamanetworkopen.2024.1838)
Supplement: Supplement 1. — eMethods. Model Specification eTable 1. Number of States Available by Year and Quarter eTable 2. ICD-10-CM Study Definitions eTable 3. Characteristics of Inpatient Stays in the Prepandemic Period (January 1, 2017–March 7, 2020) and Inpatient Stays Without a COVID-19 Diagnosis in the Peripandemic Period (March 8, 2020–December 31, 2021) eTable 4. Full Regression Models Comparing In-Hospital Mortality Among Stays During March 8–December 31, 2020, Overall and by COVID-19 Burden in the Hospital’s Community, Relative to Prepandemic Stays eTable 5. Full Regression Models Comparing In-Hospital Mortality Among Non–COVID-19 Stays in 2020 and 2021, by Month, Relative to Prepandemic Stays eFigure 1. Odds of In-Hospital Mortality Among Non–COVID-19 Stays for GI Hemorrhage, Hip Fracture, and Stroke During March 8–December 31, 2020, Overall and by COVID-19 Burden in the Hospital’s Community, Relative to Prepandemic Stays eFigure 2. Odds of In-Hospital Mortality Among Non–COVID-19 Stays for GI Hemorrhage, Hip Fracture, and Stroke in 2020 and 2021, by Month, Relative to Prepandemic Stays eFigure 3. Percentage of Hospitals in Counties With High Community COVID-19 Burden (100+ Cases per 100 000 Population in the Past 7 Days), 2020-2021 eReferences [file jamanetwopen-e241838-s001.pdf]

Supplemental Online Content

Jiang HJ, Henke RM, Fingar KR, Liang L, Agniel D. Mortality for time-sensitive conditions at urban vs rural hospitals during the COVID-19 pandemic. *JAMA Netw Open*. 2024;7(3):e241838. doi:10.1001/jamanetworkopen.2024.1838

eMethods. Model Specification

eTable 1. Number of States Available by Year and Quarter

eTable 2. *ICD-10-CM* Study Definitions

eTable 3. Characteristics of Inpatient Stays in the Prepandemic Period (January 1, 2017–March 7, 2020) and Inpatient Stays Without a Covid-19 Diagnosis in the Peri-pandemic Period (March 8, 2020–December 31, 2021)

eTable 4. Full Regression Models Comparing In-Hospital Mortality Among Stays During March 8–December 31, 2020, Overall and by Covid-19 Burden in the Hospital's Community, Relative to Prepandemic Stays

eTable 5. Full Regression Models Comparing In-Hospital Mortality Among Non–Covid-19 Stays in 2020 and 2021, by Month, Relative to Prepandemic stays

eFigure 1. Odds of In-Hospital Mortality Among Non–Covid-19 Stays for GI Hemorrhage, Hip Fracture, and Stroke During March 8–December 31, 2020, Overall and by Covid-19 Burden in the Hospital's Community, Relative to Prepandemic Stays

eFigure 2. Odds of In-Hospital Mortality Among Non-Covid-19 Stays for GI Hemorrhage, Hip Fracture, and Stroke in 2020 and 2021, by Month, Relative to Prepandemic Stays

eFigure 3. Percentage of Hospitals in Counties with High Community COVID-19 Burden (100+ Cases per 100,000 Population in the Past 7 Days), 2020-2021

eReferences

This supplemental material has been provided by the authors to give readers additional information about their work.

© 2024 Jiang HJ et al. *JAMA Network Open*.

eMethods. Model Specification

This study examined in-hospital mortality for six conditions defined using the principal diagnosis by the Clinical Classifications Software Refined (CCSR) v2022.1: acute myocardial infarction (AMI) (CIR009), gastrointestinal (GI) hemorrhage (DIG021), hip fracture (INJ006), pneumonia (RSP002), sepsis (INF002), and stroke (CIR020).[1] Covid-19 was also defined by the CCSR (INF012) using any-listed diagnoses.[1] The specific codes are listed in eTable 2.

The analysis used an interrupted time-series logistic regression model specified as follows:

$$\boldsymbol{logit(died_{it}) = \beta_0 + \beta_1 Rural + \beta_2 t + \beta_3 t{*}Rural + \beta_{4m} + \beta_5 X + \beta_6 X{*}Rural}$$

where $\mathbf{died_{it}}$ is the probability of death for person i at time t. The model included a term for rural status of the hospital, defined as being in a ZIP Code eligible for funding from the Federal Office of Rural Health Policy.[2] It included a continuous measure of time ***t*** with values 1 through 60*,* defined as the month of discharge where *t*=1 for discharges in January 2017 and where the value increases by 1 per month. This accounts for the underlying pre-period trend removing seasonality effects. We interacted ***t*** with ***Rural***, allowing for rural/urban-specific pre-period slopes. The model also included $\boldsymbol{\beta_{4m}}$, a fixed effect for calendar month (12 months), which accounts for seasonality effects that are assumed constant in the pre- and peri-pandemic periods. The model included a dichotomous indicator ***X*** (0/1), with 0 for the discharge in the prepandemic period (January 1, 2017–March 7, 2020) and 1 for the peri-pandemic period of 2020 (March 8–December 31, 2020). The main effect of interest, $\boldsymbol{\beta_5}$, corresponds to the level shift in the log odds of the death rate associated with the COVID-19 pandemic, comparing inpatient stays in the peri-pandemic period of 2020 with what would have been expected based on mortality rates in the prepandemic period. We interacted ***X*** with ***Rural*** to obtain separate effects for urban hospitals (parameterized by $\boldsymbol{\beta_5}$) and rural hospitals (parameterized by $\boldsymbol{\beta_5 + \beta_6}$). Standard errors were clustered on hospital ID. The models were weighted using entropy weights[3] that were calculated separately for stays for each condition (AMI, GI hemorrhage, hip fracture, pneumonia, sepsis, stroke), aligning age, sex, and the comorbidity score, calculated using the Elixhauser Comorbidity Software Refined for ICD-10,[4] among stays in the prepandemic period with stays in the 2020 pandemic period.

To obtain separate effects during the 2020 pandemic period by Covid-19 burden in the hospital's community, we modified the model so that *X* was replaced by the COVID-19 burden variable (0/1), with

© 2024 Jiang HJ et al. *JAMA Network Open*.

value 1 for each of the four COVID burden levels (i.e., low, moderate, substantial, or high), and 0 for stays in the prepandemic period. We ran four models estimating the effects of the pandemic for each burden level separately, using burden-specific weights. Separate entropy weights were calculated aligning age, sex, and the comorbidity score for stays in the prepandemic period with peri-pandemic stays for each level of burden.

To obtain separate effects during the 2020-2021 peri-pandemic period by month, we modified the model so that *X* was replaced by the interaction of month ***m*** and the peri-pandemic indicator ***X***. Monthly effects were estimated using quarterly data. There were seven quarters from March 2020 to December 2021 during the pandemic. Separate entropy weights were calculated comparing stays in each peripandemic quarter of 2020 (3 quarters) and 2021 (all 4 quarters) with those in the prepandemic period. We then ran quarterly models generating effect estimates for each month of that quarter separately, using quarter-specific weights. The entropy and interrupted time-series models for each quarter of 2021 excluded stays in the prepandemic period if they were in states that did not contribute to the Healthcare Cost and Utilization Project (HCUP) State Inpatient Databases (SID) in that quarter (see eTable 1).

© 2024 Jiang HJ et al. *JAMA Network Open*.

eTable 1. Number of States Available by Year and Quarter

Year	Number of states	States
2017-2020	45 states plus DC	All states except AL, ID, NE, NH, OK
2021	Quarter 1 (27 states)	AZ, CA, CO, FL, GA, HI, IA, IN, KS, KY, MD, ME, MI, MN, MO, MS, MT, NC, OH, PA, SC, TX, VA, VT, WA, WI, WV
	Quarter 2 (26 states)	AZ, CA, CO, FL, GA, HI, IA, IN, KS, KY, MD, ME, MI, MN, MS, MT, NC, OH, PA, SC, TX, VA, VT, WA, WI, WV
	Quarter 3 (20 states)	AZ, CO, GA, HI, IA, IN, KS, KY, MD, MI, MN, MS, MT, NC, OH, PA, VA, VT, WI, WV
	Quarter 4 (12 states)	AZ, CO, GA, HI, IA, KS, KY, MI, MS, MT, NJ, WV

© 2024 Jiang HJ et al. *JAMA Network Open*.

eTable 2. *ICD-10-CM* Study Definitions

ICD-10-CM code	Description
**Covid-19 (INF012)**	
J1282	Pneumonia due to coronavirus disease 2019
U071	Coronavirus disease-2019 (COVID-19)
U099	Post COVID-19 condition, unspecified
**Acute myocardial infarction (CIR009)**	
I2101	ST elevation (STEMI) myocardial infarction involving left main coronary artery
I2102	ST elevation (STEMI) myocardial infarction involving left anterior descending coronary artery
I2109	ST elevation (STEMI) myocardial infarction involving other coronary artery of anterior wall
I2111	ST elevation (STEMI) myocardial infarction involving right coronary artery
I2119	ST elevation (STEMI) myocardial infarction involving other coronary artery of inferior wall
I2121	ST elevation (STEMI) myocardial infarction involving left circumflex coronary artery
I2129	ST elevation (STEMI) myocardial infarction involving other sites
I213	ST elevation (STEMI) myocardial infarction of unspecified site
I214	Non-ST elevation (NSTEMI) myocardial infarction
I219	Acute myocardial infarction, unspecified
I21A1	Myocardial infarction type 2
I21A9	Other myocardial infarction type
I220	Subsequent ST elevation (STEMI) myocardial infarction of anterior wall
I221	Subsequent ST elevation (STEMI) myocardial infarction of inferior wall
I222	Subsequent non-ST elevation (NSTEMI) myocardial infarction
I228	Subsequent ST elevation (STEMI) myocardial infarction of other sites
I229	Subsequent ST elevation (STEMI) myocardial infarction of unspecified site
**Gastrointestinal hemorrhage (DIG021)**	
I8501	Esophageal varices with bleeding
I8511	Secondary esophageal varices with bleeding
K2081	Other esophagitis with bleeding
K2091	Esophagitis, unspecified with bleeding
K2101	Gastro-esophageal reflux disease with esophagitis, with bleeding
K250	Acute gastric ulcer with hemorrhage
K252	Acute gastric ulcer with both hemorrhage and perforation
K254	Chronic or unspecified gastric ulcer with hemorrhage
K256	Chronic or unspecified gastric ulcer with both hemorrhage and perforation
K260	Acute duodenal ulcer with hemorrhage
K262	Acute duodenal ulcer with both hemorrhage and perforation
K264	Chronic or unspecified duodenal ulcer with hemorrhage
K266	Chronic or unspecified duodenal ulcer with both hemorrhage and perforation
K270	Acute peptic ulcer, site unspecified, with hemorrhage
K272	Acute peptic ulcer, site unspecified, with both hemorrhage and perforation
K274	Chronic or unspecified peptic ulcer, site unspecified, with hemorrhage
K276	Chronic or unspecified peptic ulcer, site unspecified, with both hemorrhage and perforation
K280	Acute gastrojejunal ulcer with hemorrhage
K282	Acute gastrojejunal ulcer with both hemorrhage and perforation
K284	Chronic or unspecified gastrojejunal ulcer with hemorrhage
K286	Chronic or unspecified gastrojejunal ulcer with both hemorrhage and perforation
K2901	Acute gastritis with bleeding
K2921	Alcoholic gastritis with bleeding
K2931	Chronic superficial gastritis with bleeding
K2941	Chronic atrophic gastritis with bleeding
K2951	Unspecified chronic gastritis with bleeding
K2961	Other gastritis with bleeding
K2971	Gastritis, unspecified, with bleeding

© 2024 Jiang HJ et al. *JAMA Network Open*.

ICD-10-CM code	Description
K2981	Duodenitis with bleeding
K2991	Gastroduodenitis, unspecified, with bleeding
K31811	Angiodysplasia of stomach and duodenum with bleeding
K50011	Crohns disease of small intestine with rectal bleeding
K50111	Crohns disease of large intestine with rectal bleeding
K50811	Crohns disease of both small and large intestine with rectal bleeding
K50911	Crohns disease, unspecified, with rectal bleeding
K51011	Ulcerative (chronic) pancolitis with rectal bleeding
K51211	Ulcerative (chronic) proctitis with rectal bleeding
K51311	Ulcerative (chronic) rectosigmoiditis with rectal bleeding
K51411	Inflammatory polyps of colon with rectal bleeding
K51511	Left sided colitis with rectal bleeding
K51811	Other ulcerative colitis with rectal bleeding
K51911	Ulcerative colitis, unspecified with rectal bleeding
K5521	Angiodysplasia of colon with hemorrhage
K5701	Diverticulitis of small intestine with perforation and abscess with bleeding
K5711	Diverticulosis of small intestine without perforation or abscess with bleeding
K5713	Diverticulitis of small intestine without perforation or abscess with bleeding
K5721	Diverticulitis of large intestine with perforation and abscess with bleeding
K5731	Diverticulosis of large intestine without perforation or abscess with bleeding
K5733	Diverticulitis of large intestine without perforation or abscess with bleeding
K5741	Diverticulitis of both small and large intestine with perforation and abscess with bleeding
K5751	Diverticulosis of both small and large intestine without perforation or abscess with bleeding
K5753	Diverticulitis of both small and large intestine without perforation or abscess with bleeding
K5781	Diverticulitis of intestine, part unspecified, with perforation and abscess with bleeding
K5791	Diverticulosis of intestine, part unspecified, without perforation or abscess with bleeding
K5793	Diverticulitis of intestine, part unspecified, without perforation or abscess with bleeding
K625	Hemorrhage of anus and rectum
K920	Hematemesis
K921	Melena
K922	Gastrointestinal hemorrhage, unspecified
**Hip fracture (INJ006)**	
M9701XA	Periprosthetic fracture around internal prosthetic right hip joint, initial encounter
M9702XA	Periprosthetic fracture around internal prosthetic left hip joint, initial encounter
S72001A	Fracture of unspecified part of neck of right femur, initial encounter for closed fracture
S72001B	Fracture of unspecified part of neck of right femur, initial encounter for open fracture type I or II
S72001C	Fracture of unspecified part of neck of right femur, initial encounter for open fracture type IIIA, IIIB, or IIIC
S72002A	Fracture of unspecified part of neck of left femur, initial encounter for closed fracture
S72002B	Fracture of unspecified part of neck of left femur, initial encounter for open fracture type I or II
S72002C	Fracture of unspecified part of neck of left femur, initial encounter for open fracture type IIIA, IIIB, or IIIC
S72009A	Fracture of unspecified part of neck of unspecified femur, initial encounter for closed fracture
S72009B	Fracture of unspecified part of neck of unspecified femur, initial encounter for open fracture type I or II
S72009C	Fracture of unspecified part of neck of unspecified femur, initial encounter for open fracture type IIIA, IIIB, or IIIC
S72011A	Unspecified intracapsular fracture of right femur, initial encounter for closed fracture
S72011B	Unspecified intracapsular fracture of right femur, initial encounter for open fracture type I or II
S72011C	Unspecified intracapsular fracture of right femur, initial encounter for open fracture type IIIA, IIIB, or IIIC
S72012A	Unspecified intracapsular fracture of left femur, initial encounter for closed fracture

© 2024 Jiang HJ et al. *JAMA Network Open*.

ICD-10-CM code	Description
S72012B	Unspecified intracapsular fracture of left femur, initial encounter for open fracture type I or II
S72012C	Unspecified intracapsular fracture of left femur, initial encounter for open fracture type IIIA, IIIB, or IIIC
S72019A	Unspecified intracapsular fracture of unspecified femur, initial encounter for closed fracture
S72019B	Unspecified intracapsular fracture of unspecified femur, initial encounter for open fracture type I or II
S72019C	Unspecified intracapsular fracture of unspecified femur, initial encounter for open fracture type IIIA, IIIB, or IIIC
S72021A	Displaced fracture of epiphysis (separation) (upper) of right femur, initial encounter for closed fracture
S72021B	Displaced fracture of epiphysis (separation) (upper) of right femur, initial encounter for open fracture type I or II
S72021C	Displaced fracture of epiphysis (separation) (upper) of right femur, initial encounter for open fracture type IIIA, IIIB, or IIIC
S72022A	Displaced fracture of epiphysis (separation) (upper) of left femur, initial encounter for closed fracture
S72022B	Displaced fracture of epiphysis (separation) (upper) of left femur, initial encounter for open fracture type I or II
S72022C	Displaced fracture of epiphysis (separation) (upper) of left femur, initial encounter for open fracture type IIIA, IIIB, or IIIC
S72023A	Displaced fracture of epiphysis (separation) (upper) of unspecified femur, initial encounter for closed fracture
S72023B	Displaced fracture of epiphysis (separation) (upper) of unspecified femur, initial encounter for open fracture type I or II
S72023C	Displaced fracture of epiphysis (separation) (upper) of unspecified femur, initial encounter for open fracture type IIIA, IIIB, or IIIC
S72024A	Nondisplaced fracture of epiphysis (separation) (upper) of right femur, initial encounter for closed fracture
S72024B	Nondisplaced fracture of epiphysis (separation) (upper) of right femur, initial encounter for open fracture type I or II
S72024C	Nondisplaced fracture of epiphysis (separation) (upper) of right femur, initial encounter for open fracture type IIIA, IIIB, or IIIC
S72025A	Nondisplaced fracture of epiphysis (separation) (upper) of left femur, initial encounter for closed fracture
S72025B	Nondisplaced fracture of epiphysis (separation) (upper) of left femur, initial encounter for open fracture type I or II
S72025C	Nondisplaced fracture of epiphysis (separation) (upper) of left femur, initial encounter for open fracture type IIIA, IIIB, or IIIC
S72026A	Nondisplaced fracture of epiphysis (separation) (upper) of unspecified femur, initial encounter for closed fracture
S72026B	Nondisplaced fracture of epiphysis (separation) (upper) of unspecified femur, initial encounter for open fracture type I or II
S72026C	Nondisplaced fracture of epiphysis (separation) (upper) of unspecified femur, initial encounter for open fracture type IIIA, IIIB, or IIIC
S72031A	Displaced midcervical fracture of right femur, initial encounter for closed fracture
S72031B	Displaced midcervical fracture of right femur, initial encounter for open fracture type I or II
S72031C	Displaced midcervical fracture of right femur, initial encounter for open fracture type IIIA, IIIB, or IIIC
S72032A	Displaced midcervical fracture of left femur, initial encounter for closed fracture
S72032B	Displaced midcervical fracture of left femur, initial encounter for open fracture type I or II
S72032C	Displaced midcervical fracture of left femur, initial encounter for open fracture type IIIA, IIIB, or IIIC
S72033A	Displaced midcervical fracture of unspecified femur, initial encounter for closed fracture

© 2024 Jiang HJ et al. *JAMA Network Open*.

ICD-10-CM code	Description
S72033B	Displaced midcervical fracture of unspecified femur, initial encounter for open fracture type I or II
S72033C	Displaced midcervical fracture of unspecified femur, initial encounter for open fracture type IIIA, IIIB, or IIIC
S72034A	Nondisplaced midcervical fracture of right femur, initial encounter for closed fracture
S72034B	Nondisplaced midcervical fracture of right femur, initial encounter for open fracture type I or II
S72034C	Nondisplaced midcervical fracture of right femur, initial encounter for open fracture type IIIA, IIIB, or IIIC
S72035A	Nondisplaced midcervical fracture of left femur, initial encounter for closed fracture
S72035B	Nondisplaced midcervical fracture of left femur, initial encounter for open fracture type I or II
S72035C	Nondisplaced midcervical fracture of left femur, initial encounter for open fracture type IIIA, IIIB, or IIIC
S72036A	Nondisplaced midcervical fracture of unspecified femur, initial encounter for closed fracture
S72036B	Nondisplaced midcervical fracture of unspecified femur, initial encounter for open fracture type I or II
S72036C	Nondisplaced midcervical fracture of unspecified femur, initial encounter for open fracture type IIIA, IIIB, or IIIC
S72041A	Displaced fracture of base of neck of right femur, initial encounter for closed fracture
S72041B	Displaced fracture of base of neck of right femur, initial encounter for open fracture type I or II
S72041C	Displaced fracture of base of neck of right femur, initial encounter for open fracture type IIIA, IIIB, or IIIC
S72042A	Displaced fracture of base of neck of left femur, initial encounter for closed fracture
S72042B	Displaced fracture of base of neck of left femur, initial encounter for open fracture type I or II
S72042C	Displaced fracture of base of neck of left femur, initial encounter for open fracture type IIIA, IIIB, or IIIC
S72043A	Displaced fracture of base of neck of unspecified femur, initial encounter for closed fracture
S72043B	Displaced fracture of base of neck of unspecified femur, initial encounter for open fracture type I or II
S72043C	Displaced fracture of base of neck of unspecified femur, initial encounter for open fracture type IIIA, IIIB, or IIIC
S72044A	Nondisplaced fracture of base of neck of right femur, initial encounter for closed fracture
S72044B	Nondisplaced fracture of base of neck of right femur, initial encounter for open fracture type I or II
S72044C	Nondisplaced fracture of base of neck of right femur, initial encounter for open fracture type IIIA, IIIB, or IIIC
S72045A	Nondisplaced fracture of base of neck of left femur, initial encounter for closed fracture
S72045B	Nondisplaced fracture of base of neck of left femur, initial encounter for open fracture type I or II
S72045C	Nondisplaced fracture of base of neck of left femur, initial encounter for open fracture type IIIA, IIIB, or IIIC
S72046A	Nondisplaced fracture of base of neck of unspecified femur, initial encounter for closed fracture
S72046B	Nondisplaced fracture of base of neck of unspecified femur, initial encounter for open fracture type I or II
S72046C	Nondisplaced fracture of base of neck of unspecified femur, initial encounter for open fracture type IIIA, IIIB, or IIIC
S72051A	Unspecified fracture of head of right femur, initial encounter for closed fracture
S72051B	Unspecified fracture of head of right femur, initial encounter for open fracture type I or II
S72051C	Unspecified fracture of head of right femur, initial encounter for open fracture type IIIA, IIIB, or IIIC
S72052A	Unspecified fracture of head of left femur, initial encounter for closed fracture
S72052B	Unspecified fracture of head of left femur, initial encounter for open fracture type I or II

© 2024 Jiang HJ et al. *JAMA Network Open*.

ICD-10-CM code	Description
S72052C	Unspecified fracture of head of left femur, initial encounter for open fracture type IIIA, IIIB, or IIIC
S72059A	Unspecified fracture of head of unspecified femur, initial encounter for closed fracture
S72059B	Unspecified fracture of head of unspecified femur, initial encounter for open fracture type I or II
S72059C	Unspecified fracture of head of unspecified femur, initial encounter for open fracture type IIIA, IIIB, or IIIC
S72061A	Displaced articular fracture of head of right femur, initial encounter for closed fracture
S72061B	Displaced articular fracture of head of right femur, initial encounter for open fracture type I or II
S72061C	Displaced articular fracture of head of right femur, initial encounter for open fracture type IIIA, IIIB, or IIIC
S72062A	Displaced articular fracture of head of left femur, initial encounter for closed fracture
S72062B	Displaced articular fracture of head of left femur, initial encounter for open fracture type I or II
S72062C	Displaced articular fracture of head of left femur, initial encounter for open fracture type IIIA, IIIB, or IIIC
S72063A	Displaced articular fracture of head of unspecified femur, initial encounter for closed fracture
S72063B	Displaced articular fracture of head of unspecified femur, initial encounter for open fracture type I or II
S72063C	Displaced articular fracture of head of unspecified femur, initial encounter for open fracture type IIIA, IIIB, or IIIC
S72064A	Nondisplaced articular fracture of head of right femur, initial encounter for closed fracture
S72064B	Nondisplaced articular fracture of head of right femur, initial encounter for open fracture type I or II
S72064C	Nondisplaced articular fracture of head of right femur, initial encounter for open fracture type IIIA, IIIB, or IIIC
S72065A	Nondisplaced articular fracture of head of left femur, initial encounter for closed fracture
S72065B	Nondisplaced articular fracture of head of left femur, initial encounter for open fracture type I or II
S72065C	Nondisplaced articular fracture of head of left femur, initial encounter for open fracture type IIIA, IIIB, or IIIC
S72066A	Nondisplaced articular fracture of head of unspecified femur, initial encounter for closed fracture
S72066B	Nondisplaced articular fracture of head of unspecified femur, initial encounter for open fracture type I or II
S72066C	Nondisplaced articular fracture of head of unspecified femur, initial encounter for open fracture type IIIA, IIIB, or IIIC
S72091A	Other fracture of head and neck of right femur, initial encounter for closed fracture
S72091B	Other fracture of head and neck of right femur, initial encounter for open fracture type I or II
S72091C	Other fracture of head and neck of right femur, initial encounter for open fracture type IIIA, IIIB, or IIIC
S72092A	Other fracture of head and neck of left femur, initial encounter for closed fracture
S72092B	Other fracture of head and neck of left femur, initial encounter for open fracture type I or II
S72092C	Other fracture of head and neck of left femur, initial encounter for open fracture type IIIA, IIIB, or IIIC
S72099A	Other fracture of head and neck of unspecified femur, initial encounter for closed fracture
S72099B	Other fracture of head and neck of unspecified femur, initial encounter for open fracture type I or II
S72099C	Other fracture of head and neck of unspecified femur, initial encounter for open fracture type IIIA, IIIB, or IIIC
S72101A	Unspecified trochanteric fracture of right femur, initial encounter for closed fracture
S72101B	Unspecified trochanteric fracture of right femur, initial encounter for open fracture type I or II

© 2024 Jiang HJ et al. *JAMA Network Open*.

ICD-10-CM code	Description
S72101C	Unspecified trochanteric fracture of right femur, initial encounter for open fracture type IIIA, IIIB, or IIIC
S72102A	Unspecified trochanteric fracture of left femur, initial encounter for closed fracture
S72102B	Unspecified trochanteric fracture of left femur, initial encounter for open fracture type I or II
S72102C	Unspecified trochanteric fracture of left femur, initial encounter for open fracture type IIIA, IIIB, or IIIC
S72109A	Unspecified trochanteric fracture of unspecified femur, initial encounter for closed fracture
S72109B	Unspecified trochanteric fracture of unspecified femur, initial encounter for open fracture type I or II
S72109C	Unspecified trochanteric fracture of unspecified femur, initial encounter for open fracture type IIIA, IIIB, or IIIC
S72111A	Displaced fracture of greater trochanter of right femur, initial encounter for closed fracture
S72111B	Displaced fracture of greater trochanter of right femur, initial encounter for open fracture type I or II
S72111C	Displaced fracture of greater trochanter of right femur, initial encounter for open fracture type IIIA, IIIB, or IIIC
S72112A	Displaced fracture of greater trochanter of left femur, initial encounter for closed fracture
S72112B	Displaced fracture of greater trochanter of left femur, initial encounter for open fracture type I or II
S72112C	Displaced fracture of greater trochanter of left femur, initial encounter for open fracture type IIIA, IIIB, or IIIC
S72113A	Displaced fracture of greater trochanter of unspecified femur, initial encounter for closed fracture
S72113B	Displaced fracture of greater trochanter of unspecified femur, initial encounter for open fracture type I or II
S72113C	Displaced fracture of greater trochanter of unspecified femur, initial encounter for open fracture type IIIA, IIIB, or IIIC
S72114A	Nondisplaced fracture of greater trochanter of right femur, initial encounter for closed fracture
S72114B	Nondisplaced fracture of greater trochanter of right femur, initial encounter for open fracture type I or II
S72114C	Nondisplaced fracture of greater trochanter of right femur, initial encounter for open fracture type IIIA, IIIB, or IIIC
S72115A	Nondisplaced fracture of greater trochanter of left femur, initial encounter for closed fracture
S72115B	Nondisplaced fracture of greater trochanter of left femur, initial encounter for open fracture type I or II
S72115C	Nondisplaced fracture of greater trochanter of left femur, initial encounter for open fracture type IIIA, IIIB, or IIIC
S72116A	Nondisplaced fracture of greater trochanter of unspecified femur, initial encounter for closed fracture
S72116B	Nondisplaced fracture of greater trochanter of unspecified femur, initial encounter for open fracture type I or II
S72116C	Nondisplaced fracture of greater trochanter of unspecified femur, initial encounter for open fracture type IIIA, IIIB, or IIIC
S72121A	Displaced fracture of lesser trochanter of right femur, initial encounter for closed fracture
S72121B	Displaced fracture of lesser trochanter of right femur, initial encounter for open fracture type I or II
S72121C	Displaced fracture of lesser trochanter of right femur, initial encounter for open fracture type IIIA, IIIB, or IIIC
S72122A	Displaced fracture of lesser trochanter of left femur, initial encounter for closed fracture
S72122B	Displaced fracture of lesser trochanter of left femur, initial encounter for open fracture type I or II
S72122C	Displaced fracture of lesser trochanter of left femur, initial encounter for open fracture type IIIA, IIIB, or IIIC

© 2024 Jiang HJ et al. *JAMA Network Open*.

ICD-10-CM code	Description
S72123A	Displaced fracture of lesser trochanter of unspecified femur, initial encounter for closed fracture
S72123B	Displaced fracture of lesser trochanter of unspecified femur, initial encounter for open fracture type I or II
S72123C	Displaced fracture of lesser trochanter of unspecified femur, initial encounter for open fracture type IIIA, IIIB, or IIIC
S72124A	Nondisplaced fracture of lesser trochanter of right femur, initial encounter for closed fracture
S72124B	Nondisplaced fracture of lesser trochanter of right femur, initial encounter for open fracture type I or II
S72124C	Nondisplaced fracture of lesser trochanter of right femur, initial encounter for open fracture type IIIA, IIIB, or IIIC
S72125A	Nondisplaced fracture of lesser trochanter of left femur, initial encounter for closed fracture
S72125B	Nondisplaced fracture of lesser trochanter of left femur, initial encounter for open fracture type I or II
S72125C	Nondisplaced fracture of lesser trochanter of left femur, initial encounter for open fracture type IIIA, IIIB, or IIIC
S72126A	Nondisplaced fracture of lesser trochanter of unspecified femur, initial encounter for closed fracture
S72126B	Nondisplaced fracture of lesser trochanter of unspecified femur, initial encounter for open fracture type I or II
S72126C	Nondisplaced fracture of lesser trochanter of unspecified femur, initial encounter for open fracture type IIIA, IIIB, or IIIC
S72131A	Displaced apophyseal fracture of right femur, initial encounter for closed fracture
S72131B	Displaced apophyseal fracture of right femur, initial encounter for open fracture type I or II
S72131C	Displaced apophyseal fracture of right femur, initial encounter for open fracture type IIIA, IIIB, or IIIC
S72132A	Displaced apophyseal fracture of left femur, initial encounter for closed fracture
S72132B	Displaced apophyseal fracture of left femur, initial encounter for open fracture type I or II
S72132C	Displaced apophyseal fracture of left femur, initial encounter for open fracture type IIIA, IIIB, or IIIC
S72133A	Displaced apophyseal fracture of unspecified femur, initial encounter for closed fracture
S72133B	Displaced apophyseal fracture of unspecified femur, initial encounter for open fracture type I or II
S72133C	Displaced apophyseal fracture of unspecified femur, initial encounter for open fracture type IIIA, IIIB, or IIIC
S72134A	Nondisplaced apophyseal fracture of right femur, initial encounter for closed fracture
S72134B	Nondisplaced apophyseal fracture of right femur, initial encounter for open fracture type I or II
S72134C	Nondisplaced apophyseal fracture of right femur, initial encounter for open fracture type IIIA, IIIB, or IIIC
S72135A	Nondisplaced apophyseal fracture of left femur, initial encounter for closed fracture
S72135B	Nondisplaced apophyseal fracture of left femur, initial encounter for open fracture type I or II
S72135C	Nondisplaced apophyseal fracture of left femur, initial encounter for open fracture type IIIA, IIIB, or IIIC
S72136A	Nondisplaced apophyseal fracture of unspecified femur, initial encounter for closed fracture
S72136B	Nondisplaced apophyseal fracture of unspecified femur, initial encounter for open fracture type I or II
S72136C	Nondisplaced apophyseal fracture of unspecified femur, initial encounter for open fracture type IIIA, IIIB, or IIIC
S72141A	Displaced intertrochanteric fracture of right femur, initial encounter for closed fracture
S72141B	Displaced intertrochanteric fracture of right femur, initial encounter for open fracture type I or II
S72141C	Displaced intertrochanteric fracture of right femur, initial encounter for open fracture type IIIA, IIIB, or IIIC

© 2024 Jiang HJ et al. *JAMA Network Open*.

ICD-10-CM code	Description
S72142A	Displaced intertrochanteric fracture of left femur, initial encounter for closed fracture
S72142B	Displaced intertrochanteric fracture of left femur, initial encounter for open fracture type I or II
S72142C	Displaced intertrochanteric fracture of left femur, initial encounter for open fracture type IIIA, IIIB, or IIIC
S72143A	Displaced intertrochanteric fracture of unspecified femur, initial encounter for closed fracture
S72143B	Displaced intertrochanteric fracture of unspecified femur, initial encounter for open fracture type I or II
S72143C	Displaced intertrochanteric fracture of unspecified femur, initial encounter for open fracture type IIIA, IIIB, or IIIC
S72144A	Nondisplaced intertrochanteric fracture of right femur, initial encounter for closed fracture
S72144B	Nondisplaced intertrochanteric fracture of right femur, initial encounter for open fracture type I or II
S72144C	Nondisplaced intertrochanteric fracture of right femur, initial encounter for open fracture type IIIA, IIIB, or IIIC
S72145A	Nondisplaced intertrochanteric fracture of left femur, initial encounter for closed fracture
S72145B	Nondisplaced intertrochanteric fracture of left femur, initial encounter for open fracture type I or II
S72145C	Nondisplaced intertrochanteric fracture of left femur, initial encounter for open fracture type IIIA, IIIB, or IIIC
S72146A	Nondisplaced intertrochanteric fracture of unspecified femur, initial encounter for closed fracture
S72146B	Nondisplaced intertrochanteric fracture of unspecified femur, initial encounter for open fracture type I or II
S72146C	Nondisplaced intertrochanteric fracture of unspecified femur, initial encounter for open fracture type IIIA, IIIB, or IIIC
S7221XA	Displaced subtrochanteric fracture of right femur, initial encounter for closed fracture
S7221XB	Displaced subtrochanteric fracture of right femur, initial encounter for open fracture type I or II
S7221XC	Displaced subtrochanteric fracture of right femur, initial encounter for open fracture type IIIA, IIIB, or IIIC
S7222XA	Displaced subtrochanteric fracture of left femur, initial encounter for closed fracture
S7222XB	Displaced subtrochanteric fracture of left femur, initial encounter for open fracture type I or II
S7222XC	Displaced subtrochanteric fracture of left femur, initial encounter for open fracture type IIIA, IIIB, or IIIC
S7223XA	Displaced subtrochanteric fracture of unspecified femur, initial encounter for closed fracture
S7223XB	Displaced subtrochanteric fracture of unspecified femur, initial encounter for open fracture type I or II
S7223XC	Displaced subtrochanteric fracture of unspecified femur, initial encounter for open fracture type IIIA, IIIB, or IIIC
S7224XA	Nondisplaced subtrochanteric fracture of right femur, initial encounter for closed fracture
S7224XB	Nondisplaced subtrochanteric fracture of right femur, initial encounter for open fracture type I or II
S7224XC	Nondisplaced subtrochanteric fracture of right femur, initial encounter for open fracture type IIIA, IIIB, or IIIC
S7225XA	Nondisplaced subtrochanteric fracture of left femur, initial encounter for closed fracture
S7225XB	Nondisplaced subtrochanteric fracture of left femur, initial encounter for open fracture type I or II
S7225XC	Nondisplaced subtrochanteric fracture of left femur, initial encounter for open fracture type IIIA, IIIB, or IIIC
S7226XA	Nondisplaced subtrochanteric fracture of unspecified femur, initial encounter for closed fracture
S7226XB	Nondisplaced subtrochanteric fracture of unspecified femur, initial encounter for open fracture type I or II

© 2024 Jiang HJ et al. *JAMA Network Open*.

ICD-10-CM code	Description
S7226XC	Nondisplaced subtrochanteric fracture of unspecified femur, initial encounter for open fracture type IIIA, IIIB, or IIIC
T84040A	Periprosthetic fracture around internal prosthetic right hip joint, initial encounter
T84041A	Periprosthetic fracture around internal prosthetic left hip joint, initial encounter
**Pneumonia (RSP002)**	
A0103	Typhoid pneumonia
A0222	Salmonella pneumonia
A202	Pneumonic plague
A212	Pulmonary tularemia
A221	Pulmonary anthrax
A310	Pulmonary mycobacterial infection
A3701	Whooping cough due to Bordetella pertussis with pneumonia
A3711	Whooping cough due to Bordetella parapertussis with pneumonia
A3781	Whooping cough due to other Bordetella species with pneumonia
A3791	Whooping cough, unspecified species with pneumonia
A430	Pulmonary nocardiosis
A481	Legionnaires disease
A5004	Early congenital syphilitic pneumonia
A5272	Syphilis of lung and bronchus
A5484	Gonococcal pneumonia
B012	Varicella pneumonia
B052	Measles complicated by pneumonia
B0681	Rubella pneumonia
B250	Cytomegaloviral pneumonitis
B371	Pulmonary candidiasis
B380	Acute pulmonary coccidioidomycosis
B381	Chronic pulmonary coccidioidomycosis
B382	Pulmonary coccidioidomycosis, unspecified
B390	Acute pulmonary histoplasmosis capsulati
B391	Chronic pulmonary histoplasmosis capsulati
B392	Pulmonary histoplasmosis capsulati, unspecified
B583	Pulmonary toxoplasmosis
B59	Pneumocystosis
B7781	Ascariasis pneumonia
J09X1	Influenza due to identified novel influenza A virus with pneumonia
J1000	Influenza due to other identified influenza virus with unspecified type of pneumonia
J1001	Influenza due to other identified influenza virus with the same other identified influenza virus pneumonia
J1008	Influenza due to other identified influenza virus with other specified pneumonia
J1100	Influenza due to unidentified influenza virus with unspecified type of pneumonia
J1108	Influenza due to unidentified influenza virus with specified pneumonia
J120	Adenoviral pneumonia
J121	Respiratory syncytial virus pneumonia
J122	Parainfluenza virus pneumonia
J123	Human metapneumovirus pneumonia
J1281	Pneumonia due to SARS-associated coronavirus
J1282	Pneumonia due to coronavirus disease 2019
J1289	Other viral pneumonia
J129	Viral pneumonia, unspecified
J13	Pneumonia due to Streptococcus pneumoniae
J14	Pneumonia due to Hemophilus influenzae
J150	Pneumonia due to Klebsiella pneumoniae
J151	Pneumonia due to Pseudomonas

© 2024 Jiang HJ et al. *JAMA Network Open*.

ICD-10-CM code	Description
J1520	Pneumonia due to staphylococcus, unspecified
J15211	Pneumonia due to Methicillin susceptible Staphylococcus aureus
J15212	Pneumonia due to Methicillin resistant Staphylococcus aureus
J1529	Pneumonia due to other staphylococcus
J153	Pneumonia due to streptococcus, group B
J154	Pneumonia due to other streptococci
J155	Pneumonia due to Escherichia coli
J156	Pneumonia due to other Gram-negative bacteria
J157	Pneumonia due to Mycoplasma pneumoniae
J158	Pneumonia due to other specified bacteria
J159	Unspecified bacterial pneumonia
J160	Chlamydial pneumonia
J168	Pneumonia due to other specified infectious organisms
J17	Pneumonia in diseases classified elsewhere
J180	Bronchopneumonia, unspecified organism
J181	Lobar pneumonia, unspecified organism
J188	Other pneumonia, unspecified organism
J189	Pneumonia, unspecified organism
J851	Abscess of lung with pneumonia
J95851	Ventilator associated pneumonia
**Sepsis (INF002)**	
A021	Salmonella sepsis
A207	Septicemic plague
A227	Anthrax sepsis
A267	Erysipelothrix sepsis
A327	Listerial sepsis
A392	Acute meningococcemia
A393	Chronic meningococcemia
A394	Meningococcemia, unspecified
A400	Sepsis due to streptococcus, group A
A401	Sepsis due to streptococcus, group B
A403	Sepsis due to Streptococcus pneumoniae
A408	Other streptococcal sepsis
A409	Streptococcal sepsis, unspecified
A4101	Sepsis due to Methicillin susceptible Staphylococcus aureus
A4102	Sepsis due to Methicillin resistant Staphylococcus aureus
A411	Sepsis due to other specified staphylococcus
A412	Sepsis due to unspecified staphylococcus
A413	Sepsis due to Hemophilus influenzae
A414	Sepsis due to anaerobes
A4150	Gram-negative sepsis, unspecified
A4151	Sepsis due to Escherichia coli [E. coli]
A4152	Sepsis due to Pseudomonas
A4153	Sepsis due to Serratia
A4159	Other Gram-negative sepsis
A4181	Sepsis due to Enterococcus
A4189	Other specified sepsis
A419	Sepsis, unspecified organism
A427	Actinomycotic sepsis
A5486	Gonococcal sepsis
B007	Disseminated herpesviral disease
B377	Candidal sepsis

© 2024 Jiang HJ et al. *JAMA Network Open*.

ICD-10-CM code	Description
I76	Septic arterial embolism
O0337	Sepsis following incomplete spontaneous abortion
O0387	Sepsis following complete or unspecified spontaneous abortion
O0487	Sepsis following (induced) termination of pregnancy
O0737	Sepsis following failed attempted termination of pregnancy
O0882	Sepsis following ectopic and molar pregnancy
O85	Puerperal sepsis
O8604	Sepsis following an obstetrical procedure
P360	Sepsis of newborn due to streptococcus, group B
P3610	Sepsis of newborn due to unspecified streptococci
P3619	Sepsis of newborn due to other streptococci
P362	Sepsis of newborn due to Staphylococcus aureus
P3630	Sepsis of newborn due to unspecified staphylococci
P3639	Sepsis of newborn due to other staphylococci
P364	Sepsis of newborn due to Escherichia coli
P365	Sepsis of newborn due to anaerobes
P368	Other bacterial sepsis of newborn
P369	Bacterial sepsis of newborn, unspecified
R6520	Severe sepsis without septic shock
R6521	Severe sepsis with septic shock
T8112XA	Postprocedural septic shock, initial encounter
T8144XA	Sepsis following a procedure, initial encounter
**Stroke (CIR020)**	
G43601	Persistent migraine aura with cerebral infarction, not intractable, with status migrainosus
G43609	Persistent migraine aura with cerebral infarction, not intractable, without status migrainosus
G43611	Persistent migraine aura with cerebral infarction, intractable, with status migrainosus
G43619	Persistent migraine aura with cerebral infarction, intractable, without status migrainosus
I6300	Cerebral infarction due to thrombosis of unspecified precerebral artery
I63011	Cerebral infarction due to thrombosis of right vertebral artery
I63012	Cerebral infarction due to thrombosis of left vertebral artery
I63013	Cerebral infarction due to thrombosis of bilateral vertebral arteries
I63019	Cerebral infarction due to thrombosis of unspecified vertebral artery
I6302	Cerebral infarction due to thrombosis of basilar artery
I63031	Cerebral infarction due to thrombosis of right carotid artery
I63032	Cerebral infarction due to thrombosis of left carotid artery
I63033	Cerebral infarction due to thrombosis of bilateral carotid arteries
I63039	Cerebral infarction due to thrombosis of unspecified carotid artery
I6309	Cerebral infarction due to thrombosis of other precerebral artery
I6310	Cerebral infarction due to embolism of unspecified precerebral artery
I63111	Cerebral infarction due to embolism of right vertebral artery
I63112	Cerebral infarction due to embolism of left vertebral artery
I63113	Cerebral infarction due to embolism of bilateral vertebral arteries
I63119	Cerebral infarction due to embolism of unspecified vertebral artery
I6312	Cerebral infarction due to embolism of basilar artery
I63131	Cerebral infarction due to embolism of right carotid artery
I63132	Cerebral infarction due to embolism of left carotid artery
I63133	Cerebral infarction due to embolism of bilateral carotid arteries
I63139	Cerebral infarction due to embolism of unspecified carotid artery
I6319	Cerebral infarction due to embolism of other precerebral artery
I6320	Cerebral infarction due to unspecified occlusion or stenosis of unspecified precerebral arteries
I63211	Cerebral infarction due to unspecified occlusion or stenosis of right vertebral artery
I63212	Cerebral infarction due to unspecified occlusion or stenosis of left vertebral artery

© 2024 Jiang HJ et al. *JAMA Network Open*.

ICD-10-CM code	Description
I63213	Cerebral infarction due to unspecified occlusion or stenosis of bilateral vertebral arteries
I63219	Cerebral infarction due to unspecified occlusion or stenosis of unspecified vertebral artery
I6322	Cerebral infarction due to unspecified occlusion or stenosis of basilar artery
I63231	Cerebral infarction due to unspecified occlusion or stenosis of right carotid arteries
I63232	Cerebral infarction due to unspecified occlusion or stenosis of left carotid arteries
I63233	Cerebral infarction due to unspecified occlusion or stenosis of bilateral carotid arteries
I63239	Cerebral infarction due to unspecified occlusion or stenosis of unspecified carotid artery
I6329	Cerebral infarction due to unspecified occlusion or stenosis of other precerebral arteries
I6330	Cerebral infarction due to thrombosis of unspecified cerebral artery
I63311	Cerebral infarction due to thrombosis of right middle cerebral artery
I63312	Cerebral infarction due to thrombosis of left middle cerebral artery
I63313	Cerebral infarction due to thrombosis of bilateral middle cerebral arteries
I63319	Cerebral infarction due to thrombosis of unspecified middle cerebral artery
I63321	Cerebral infarction due to thrombosis of right anterior cerebral artery
I63322	Cerebral infarction due to thrombosis of left anterior cerebral artery
I63323	Cerebral infarction due to thrombosis of bilateral anterior cerebral arteries
I63329	Cerebral infarction due to thrombosis of unspecified anterior cerebral artery
I63331	Cerebral infarction due to thrombosis of right posterior cerebral artery
I63332	Cerebral infarction due to thrombosis of left posterior cerebral artery
I63333	Cerebral infarction due to thrombosis of bilateral posterior cerebral arteries
I63339	Cerebral infarction due to thrombosis of unspecified posterior cerebral artery
I63341	Cerebral infarction due to thrombosis of right cerebellar artery
I63342	Cerebral infarction due to thrombosis of left cerebellar artery
I63343	Cerebral infarction due to thrombosis of bilateral cerebellar arteries
I63349	Cerebral infarction due to thrombosis of unspecified cerebellar artery
I6339	Cerebral infarction due to thrombosis of other cerebral artery
I6340	Cerebral infarction due to embolism of unspecified cerebral artery
I63411	Cerebral infarction due to embolism of right middle cerebral artery
I63412	Cerebral infarction due to embolism of left middle cerebral artery
I63413	Cerebral infarction due to embolism of bilateral middle cerebral arteries
I63419	Cerebral infarction due to embolism of unspecified middle cerebral artery
I63421	Cerebral infarction due to embolism of right anterior cerebral artery
I63422	Cerebral infarction due to embolism of left anterior cerebral artery
I63423	Cerebral infarction due to embolism of bilateral anterior cerebral arteries
I63429	Cerebral infarction due to embolism of unspecified anterior cerebral artery
I63431	Cerebral infarction due to embolism of right posterior cerebral artery
I63432	Cerebral infarction due to embolism of left posterior cerebral artery
I63433	Cerebral infarction due to embolism of bilateral posterior cerebral arteries
I63439	Cerebral infarction due to embolism of unspecified posterior cerebral artery
I63441	Cerebral infarction due to embolism of right cerebellar artery
I63442	Cerebral infarction due to embolism of left cerebellar artery
I63443	Cerebral infarction due to embolism of bilateral cerebellar arteries
I63449	Cerebral infarction due to embolism of unspecified cerebellar artery
I6349	Cerebral infarction due to embolism of other cerebral artery
I6350	Cerebral infarction due to unspecified occlusion or stenosis of unspecified cerebral artery
I63511	Cerebral infarction due to unspecified occlusion or stenosis of right middle cerebral artery
I63512	Cerebral infarction due to unspecified occlusion or stenosis of left middle cerebral artery
I63513	Cerebral infarction due to unspecified occlusion or stenosis of bilateral middle cerebral arteries
I63519	Cerebral infarction due to unspecified occlusion or stenosis of unspecified middle cerebral artery
I63521	Cerebral infarction due to unspecified occlusion or stenosis of right anterior cerebral artery
I63522	Cerebral infarction due to unspecified occlusion or stenosis of left anterior cerebral artery

© 2024 Jiang HJ et al. *JAMA Network Open*.

ICD-10-CM code	Description
I63523	Cerebral infarction due to unspecified occlusion or stenosis of bilateral anterior cerebral arteries
I63529	Cerebral infarction due to unspecified occlusion or stenosis of unspecified anterior cerebral artery
I63531	Cerebral infarction due to unspecified occlusion or stenosis of right posterior cerebral artery
I63532	Cerebral infarction due to unspecified occlusion or stenosis of left posterior cerebral artery
I63533	Cerebral infarction due to unspecified occlusion or stenosis of bilateral posterior cerebral arteries
I63539	Cerebral infarction due to unspecified occlusion or stenosis of unspecified posterior cerebral artery
I63541	Cerebral infarction due to unspecified occlusion or stenosis of right cerebellar artery
I63542	Cerebral infarction due to unspecified occlusion or stenosis of left cerebellar artery
I63543	Cerebral infarction due to unspecified occlusion or stenosis of bilateral cerebellar arteries
I63549	Cerebral infarction due to unspecified occlusion or stenosis of unspecified cerebellar artery
I6359	Cerebral infarction due to unspecified occlusion or stenosis of other cerebral artery
I636	Cerebral infarction due to cerebral venous thrombosis, nonpyogenic
I638	Other cerebral infarction
I6381	Other cerebral infarction due to occlusion or stenosis of small artery
I6389	Other cerebral infarction
I639	Cerebral infarction, unspecified
I97810	Intraoperative cerebrovascular infarction during cardiac surgery
I97811	Intraoperative cerebrovascular infarction during other surgery
I97820	Postprocedural cerebrovascular infarction following cardiac surgery
I97821	Postprocedural cerebrovascular infarction following other surgery
R29700	NIHSS score 0
R29701	NIHSS score 1
R29702	NIHSS score 2
R29703	NIHSS score 3
R29704	NIHSS score 4
R29705	NIHSS score 5
R29706	NIHSS score 6
R29707	NIHSS score 7
R29708	NIHSS score 8
R29709	NIHSS score 9
R29710	NIHSS score 10
R29711	NIHSS score 11
R29712	NIHSS score 12
R29713	NIHSS score 13
R29714	NIHSS score 14
R29715	NIHSS score 15
R29716	NIHSS score 16
R29717	NIHSS score 17
R29718	NIHSS score 18
R29719	NIHSS score 19
R29720	NIHSS score 20
R29721	NIHSS score 21
R29722	NIHSS score 22
R29723	NIHSS score 23
R29724	NIHSS score 24
R29725	NIHSS score 25
R29726	NIHSS score 26
R29727	NIHSS score 27
R29728	NIHSS score 28

© 2024 Jiang HJ et al. *JAMA Network Open*.

ICD-10-CM code	Description
R29729	NIHSS score 29
R29730	NIHSS score 30
R29731	NIHSS score 31
R29732	NIHSS score 32
R29733	NIHSS score 33
R29734	NIHSS score 34
R29735	NIHSS score 35
R29736	NIHSS score 36
R29737	NIHSS score 37
R29738	NIHSS score 38
R29739	NIHSS score 39
R29740	NIHSS score 40
R29741	NIHSS score 41
R29742	NIHSS score 42

Abbreviation: ICD-10-CM, International Classification of Diseases, Tenth Revision, Clinical Modification

© 2024 Jiang HJ et al. *JAMA Network Open*.

eTable 3. Characteristics of Inpatient Stays in the Prepandemic Period (January 1, 2017–March 7, 2020) and Inpatient Stays Without a Covid-19 Diagnosis in the Peri-pandemic Period (March 8, 2020–December 31, 2021)

Characteristic	Stays for AMI			Stays for sepsis			Stays for pneumonia			Stays for GI hemorrhage			Stays for hip fracture			Stays for stroke		
	Pre	Non-Covid peri	SMD	Pre	Non-Covid peri	SMD	Pre	Non-Covid peri	SMD	Pre	Non-Covid peri	SMD	Pre	Non-Covid peri	SMD	Pre	Non-Covid peri	SMD
Total, N	1,882,182	627,168		6,523,717	2,267,617		1,935,244	517,257		984,318	308,652		981,277	370,915		1,607,018	596,560	
Age, years, %																		
18–24	0.1	0.1	0.00	2.1	2.0	0.00	1.3	1.1	0.02	0.7	0.7	0.01	0.4	0.5	-0.01	0.2	0.2	0.00
25–44	5.1	5.5	-0.02	11.7	12.8	-0.03	8.0	8.2	-0.01	7.6	7.6	0.00	1.8	2.1	-0.02	4.1	4.3	-0.01
45–64	37.7	38.5	-0.02	29.4	29.9	-0.01	25.8	27.5	-0.04	27.4	26.9	0.01	11.4	11.2	0.00	28.9	29.6	-0.02
65–84	45.6	46.1	-0.01	42.5	43.1	-0.01	45.3	47.4	-0.04	47.2	49.0	-0.04	48.9	50.6	-0.03	48.6	49.6	-0.02
85+	11.5	9.9	0.05	14.3	12.1	0.06	19.7	15.8	0.10	17.1	15.8	0.04	37.6	35.7	0.04	18.1	16.2	0.05
Female, %	37.7	36.4	0.03	50.4	49.2	0.03	53.2	50.1	0.06	48.0	47.6	0.01	67.1	66.0	0.02	49.7	49.0	0.02
Race and ethnicity, %																		
API NH	2.8	3.0	-0.01	3.1	3.0	0.01	2.1	2.0	0.01	3.3	3.5	-0.01	1.8	1.8	0.00	3.0	3.1	-0.01
Black NH	11.2	10.9	0.01	13.0	13.5	-0.01	12.0	13.4	-0.04	14.5	14.3	0.00	4.5	4.6	0.00	17.0	16.9	0.00
Hispanic	8.8	8.6	0.01	11.0	10.7	0.01	8.2	7.5	0.03	9.1	8.6	0.02	5.7	5.2	0.02	8.3	7.9	0.02
White NH	70.7	72.5	-0.04	67.0	66.5	0.01	72.1	71.0	0.03	67.7	67.4	0.01	83.0	82.9	0.00	65.9	65.4	0.01
AI/AN NH	0.6	0.5	0.01	0.8	0.8	0.00	0.7	0.6	0.01	0.7	0.8	-0.01	0.4	0.4	0.01	0.5	0.4	0.01
Other NH	3.0	2.9	0.01	2.6	2.3	0.02	2.3	1.9	0.03	2.4	2.0	0.03	2.0	1.8	0.01	2.6	2.3	0.02
Community income, %																		
Quartile 1 (lowest)	28.6	28.8	0.00	29.6	30.2	-0.01	29.9	31.1	-0.03	29.1	29.1	0.00	23.8	24.0	-0.01	28.8	28.8	0.00
Quartile 2	27.3	27.4	0.00	26.5	26.5	0.00	27.6	27.7	0.00	26.5	26.6	0.00	26.5	26.5	0.00	26.3	26.4	0.00
Quartile 3	23.7	23.6	0.00	23.5	23.2	0.01	23.1	22.8	0.01	23.5	23.6	0.00	25.2	25.2	0.00	23.8	23.9	0.00
Quartile 4 (highest)	18.6	18.7	0.00	18.5	18.1	0.01	17.7	16.8	0.02	19.2	19.0	0.00	23.0	22.9	0.00	19.5	19.4	0.00
Expected payer, %																		
Medicare	57.0	54.9	0.04	61.7	59.3	0.05	68.4	66.2	0.05	66.4	65.6	0.02	82.5	81.8	0.02	65.2	63.7	0.03
Medicaid	9.5	10.1	-0.02	14.2	15.6	-0.04	10.6	11.4	-0.03	10.8	11.3	-0.02	3.8	4.2	-0.02	9.3	10.0	-0.02
Private insurance	25.3	26.1	-0.02	17.5	17.8	-0.01	15.8	16.1	-0.01	16.4	16.3	0.00	9.8	9.7	0.00	18.8	19.0	0.00
Self-pay/no charge[a]	5.2	5.5	-0.01	4.3	4.7	-0.02	3.2	3.5	-0.01	4.1	4.2	0.00	1.5	1.6	0.00	4.4	4.6	-0.01
Other	2.8	3.3	-0.03	2.1	2.5	-0.03	1.9	2.6	-0.05	2.1	2.6	-0.03	2.3	2.6	-0.02	2.2	2.7	-0.03
Rural hospital, %	8.0	8.9	-0.03	11.2	13.1	-0.06	21.0	20.8	0.00	10.6	11.2	-0.02	13.5	14.4	-0.03	8.5	9.0	-0.02

© 2024 Jiang HJ et al. *JAMA Network Open*.

Characteristic	Stays for AMI			Stays for sepsis			Stays for pneumonia			Stays for GI hemorrhage			Stays for hip fracture			Stays for stroke		
	Pre	Non-Covid peri	SMD	Pre	Non-Covid peri	SMD	Pre	Non-Covid peri	SMD	Pre	Non-Covid peri	SMD	Pre	Non-Covid peri	SMD	Pre	Non-Covid peri	SMD
Elixhauser comorbidity index score (mean)	7.00	6.69	0.01	12.59	13.15	-0.02	8.56	9.17	-0.02	8.7	9.67	-0.03	5.73	5.84	0.00	10.18	10.95	-0.03
Comorbidities, %																		
HIV/AIDS	0.5	0.4	0.00	1.0	1.0	0.00	1.3	1.1	0.01	0.6	0.5	0.01	0.2	0.2	0.01	0.5	0.4	0.00
Alcohol abuse	3.4	3.6	-0.01	4.8	5.8	-0.05	3.0	3.8	-0.05	10.8	11.9	-0.03	4.5	4.8	-0.02	4.4	4.7	-0.02
Deficiency anemias	15.3	15.1	0.01	27.7	29.4	-0.04	23.7	25.9	-0.05	24.5	26.4	-0.04	17.9	19.0	-0.03	11.4	12.5	-0.03
Autoimmune conditions	2.9	3.0	-0.01	4.5	4.3	0.01	5.2	5.3	0.00	3.8	4.0	-0.01	4.1	4.1	0.00	3.0	3.1	-0.01
Chronic blood loss anemia	0.5	0.4	0.02	0.8	0.8	0.01	0.5	0.5	0.00	9.2	6.7	0.09	1.0	0.8	0.03	0.3	0.3	0.00
Leukemia	0.5	0.5	0.00	1.3	1.3	0.00	1.6	1.5	0.01	0.7	0.8	-0.01	0.6	0.6	0.00	0.4	0.5	0.00
Lymphoma	0.5	0.5	0.00	1.8	1.6	0.01	2.1	1.8	0.02	1.0	1.0	-0.01	0.7	0.7	0.00	0.5	0.6	-0.01
Metastatic cancer	0.9	1.0	0.00	4.9	5.5	-0.03	4.0	4.7	-0.03	3.2	3.5	-0.02	1.2	1.2	0.00	1.8	2.0	-0.02
Solid tumor without metastasis, in situ	0.0	0.0	0.00	0.0	0.0	0.00	0.0	0.0	0.00	0.0	0.0	0.00	0.0	0.0	0.00	0.0	0.0	0.00
Solid tumor without metastasis, malignant	1.8	1.8	0.00	4.7	5.0	-0.01	4.7	5.3	-0.03	4.1	4.4	-0.01	2.3	2.4	-0.01	2.1	2.3	-0.01
Cerebrovascular disease	5.2	5.2	0.00	5.5	5.6	0.00	3.6	3.7	-0.01	4.6	4.9	-0.01	5.1	5.1	0.00	29.2	32.2	-0.06
Heart failure	37.6	38.6	-0.02	24.4	25.2	-0.02	26.6	29.0	-0.05	22.4	24.9	-0.06	16.0	16.7	-0.02	16.5	17.6	-0.03
Coagulopathy	4.3	4.1	0.01	13.4	14.0	-0.02	6.6	7.0	-0.02	13.4	14.7	-0.04	6.1	6.2	0.00	3.6	3.8	-0.01
Dementia	5.6	4.9	0.03	13.1	12.0	0.03	11.2	10.4	0.02	10.0	9.6	0.01	25.6	25.4	0.01	12.0	11.1	0.03
Depression	9.2	9.8	-0.02	13.4	13.6	-0.01	14.4	15.6	-0.03	13.1	13.7	-0.02	15.4	16.2	-0.02	11.3	11.8	-0.01
Diabetes with chronic complications	26.0	27.2	-0.03	26.1	28.9	-0.06	19.6	22.0	-0.06	20.0	22.6	-0.06	13.6	15.5	-0.05	23.3	25.7	-0.06
Diabetes without chronic complications	14.7	13.8	0.03	11.4	10.3	0.04	12.4	11.7	0.02	12.3	10.7	0.05	10.0	9.1	0.03	16.0	14.6	0.04
Drug abuse	2.9	3.3	-0.02	5.3	6.4	-0.04	3.5	4.3	-0.04	3.0	3.5	-0.02	1.7	1.9	-0.02	2.8	3.3	-0.03

© 2024 Jiang HJ et al. *JAMA Network Open*.

Characteristic	Stays for AMI			Stays for sepsis			Stays for pneumonia			Stays for GI hemorrhage			Stays for hip fracture			Stays for stroke		
	Pre	Non-Covid peri	SMD	Pre	Non-Covid peri	SMD	Pre	Non-Covid peri	SMD	Pre	Non-Covid peri	SMD	Pre	Non-Covid peri	SMD	Pre	Non-Covid peri	SMD
Hypertension, complicated	43.5	44.6	-0.02	33.6	34.8	-0.02	34.1	37.2	-0.06	34.3	37.5	-0.07	27.2	28.9	-0.04	31.6	34.4	-0.06
Hypertension, uncomplicated	38.9	38.5	0.01	31.6	31.1	0.01	35.1	34.1	0.02	37.7	36.0	0.04	45.7	44.8	0.02	54.4	52.7	0.04
Liver disease, mild	2.9	3.3	-0.03	6.8	8.1	-0.05	4.2	5.0	-0.04	6.9	8.1	-0.04	2.4	2.7	-0.02	2.0	2.4	-0.02
Liver disease, moderate to severe	0.4	0.5	0.00	2.6	3.2	-0.04	0.8	1.0	-0.02	7.3	7.9	-0.02	0.6	0.6	-0.01	0.3	0.4	-0.01
Chronic pulmonary disease	21.7	20.7	0.02	29.5	26.6	0.07	44.9	47.1	-0.05	22.8	23.1	-0.01	21.8	22.2	-0.01	15.8	16.3	-0.01
Neurological disorders affecting movement	2.0	2.1	-0.01	3.6	3.6	0.00	3.6	3.9	-0.02	2.9	3.1	-0.01	5.0	5.3	-0.01	2.9	3.0	-0.01
Other neurological disorders	3.2	3.4	-0.01	17.6	20.1	-0.07	6.9	8.8	-0.07	3.6	4.7	-0.05	3.7	4.4	-0.03	16.1	18.8	-0.07
Seizures and epilepsy	2.1	2.0	0.00	5.7	5.8	0.00	3.9	4.3	-0.02	3.7	3.7	0.00	3.5	3.6	0.00	5.6	5.8	-0.01
Obesity	20.4	23.7	-0.08	18.7	22.0	-0.08	15.2	18.7	-0.09	14.0	16.6	-0.07	6.1	7.3	-0.05	14.4	17.1	-0.07
Paralysis	2.2	2.0	0.02	7.2	6.9	0.01	3.4	3.4	0.00	3.7	3.7	0.00	3.5	3.3	0.01	52.6	53.7	-0.02
Peripheral vascular disease	10.6	10.5	0.00	7.2	7.6	-0.01	6.4	6.9	-0.02	8.5	9.5	-0.03	7.3	7.7	-0.02	10.2	10.7	-0.02
Psychoses	2.1	2.5	-0.02	5.0	5.7	-0.03	4.5	5.7	-0.06	3.5	4.0	-0.03	3.0	3.5	-0.03	2.5	3.0	-0.03
Pulmonary circulation disease	5.4	5.4	0.00	4.8	4.9	0.00	6.2	6.6	-0.02	4.4	4.9	-0.02	4.4	4.5	-0.01	3.1	3.2	-0.01
Renal failure, moderate	15.2	15.2	0.00	15.5	16.1	-0.02	13.8	14.8	-0.03	15.5	17.2	-0.04	14.1	15.6	-0.04	12.9	14.0	-0.03
Renal failure, severe	7.7	7.2	0.02	8.3	8.5	-0.01	8.1	8.4	-0.01	9.7	10.5	-0.03	4.5	4.6	0.00	4.1	4.3	-0.01
Hypothyroidism	12.6	12.8	-0.01	14.9	14.8	0.00	17.5	17.5	0.00	15.2	15.9	-0.02	21.3	21.6	-0.01	14.4	14.6	-0.01
Other thyroid disorders	1.0	1.1	-0.01	1.1	1.2	-0.01	1.3	1.4	-0.01	0.9	1.0	-0.02	1.2	1.5	-0.02	2.0	2.5	-0.03

© 2024 Jiang HJ et al. *JAMA Network Open*.

Characteristic	Stays for AMI			Stays for sepsis			Stays for pneumonia			Stays for GI hemorrhage			Stays for hip fracture			Stays for stroke		
	Pre	Non-Covid peri	SMD	Pre	Non-Covid peri	SMD	Pre	Non-Covid peri	SMD	Pre	Non-Covid peri	SMD	Pre	Non-Covid peri	SMD	Pre	Non-Covid peri	SMD
Peptic ulcer disease with bleeding	0.7	0.7	0.01	1.4	1.7	-0.02	0.6	0.6	0.00	11.2	9.0	0.07	0.5	0.5	0.00	0.6	0.6	0.00
Valvular disease	14.6	14.3	0.01	7.7	7.6	0.00	8.1	8.0	0.00	9.8	10.2	-0.01	10.1	10.0	0.00	9.9	9.8	0.00
Weight loss	3.2	2.9	0.02	15.2	15.3	0.00	9.6	9.8	-0.01	9.5	10.1	-0.02	8.0	8.3	-0.01	4.4	4.6	-0.01

Abbreviations: AI/AN, American Indian/Alaska Native; AIDS, acquired immunodeficiency syndrome; AMI, acute myocardial infarction; API, Asian/Pacific Islander; GI, gastrointestinal; HIV, human immunodeficiency virus; NH, non-Hispanic; SMD, standardized mean difference.

[a] Self-pay/no charge: includes self-pay, no charge, charity, and no expected payment.

© 2024 Jiang HJ et al. *JAMA Network Open*.

eTable 4. Full Regression Models Comparing In-Hospital Mortality Among Stays During March 8–December 31, 2020, Overall and by Covid-19 Burden in the Hospital's Community, Relative to Prepandemic Stays

Reason for hospital stay	March 8–December 31, 2020, overall			Covid-19 burden											
				Low			Moderate			Substantial			High		
	OR	Lower CI	Upper CI	OR	Lower CI	Upper CI	OR	Lower CI	Upper CI	OR	Lower CI	Upper CI	OR	Lower CI	Upper CI
**Rural**															
Non-Covid-19															
AMI	1.06	0.98	1.16	1.13	0.98	1.31	1.08	0.92	1.27	1.08	0.85	1.39	0.99	0.87	1.12
Hip fracture	**1.32**	**1.14**	**1.53**	**1.37**	**1.08**	**1.73**	1.22	0.97	1.54	1.26	0.94	1.69	1.04	0.85	1.28
GI hemorrhage	1.08	0.94	1.26	0.90	0.70	1.14	1.04	0.81	1.33	1.01	0.76	1.33	1.02	0.83	1.25
Pneumonia	**1.46**	**1.36**	**1.57**	**1.30**	**1.17**	**1.44**	**1.34**	**1.17**	**1.52**	**1.62**	**1.38**	**1.89**	**1.66**	**1.48**	**1.88**
Sepsis	**1.35**	**1.30**	**1.40**	**1.22**	**1.15**	**1.29**	**1.41**	**1.32**	**1.52**	**1.46**	**1.34**	**1.59**	**1.54**	**1.44**	**1.66**
Stroke	1.05	0.95	1.17	1.07	0.90	1.26	0.98	0.80	1.18	0.96	0.78	1.19	1.03	0.88	1.21
**Urban**															
Non-Covid-19															
AMI	**1.09**	**1.06**	**1.12**	1.02	0.95	1.10	1.01	0.93	1.10	1.06	0.99	1.15	**1.16**	**1.13**	**1.20**
Hip fracture	1.05	0.99	1.11	1.08	0.92	1.27	1.17	0.99	1.38	0.97	0.83	1.14	1.06	1.00	1.13
GI hemorrhage	**1.15**	**1.09**	**1.21**	**1.16**	**1.00**	**1.33**	1.08	0.93	1.27	1.14	0.99	1.31	**1.11**	**1.05**	**1.17**
Pneumonia	**1.48**	**1.42**	**1.54**	**1.34**	**1.22**	**1.47**	**1.49**	**1.31**	**1.69**	**1.45**	**1.28**	**1.64**	**1.61**	**1.53**	**1.70**
Sepsis	**1.27**	**1.25**	**1.29**	**1.16**	**1.13**	**1.20**	**1.17**	**1.12**	**1.22**	**1.20**	**1.16**	**1.26**	**1.28**	**1.25**	**1.31**
Stroke	1.00	0.97	1.03	1.00	0.91	1.11	0.98	0.87	1.10	1.00	0.91	1.10	1.00	0.96	1.04

Abbreviations: AMI, acute myocardial infarction; CI, confident interval; GI, gastrointestinal; OR, odds ratio

Note: Values in bold are statistically significant ($p<0.05$). Odds ratio estimates are from interrupted time-series logistic regression models. Each model included the following independent variables: rural status (based on hospital location), month of discharge (continuous 1–60 starting in January 2017), calendar month (values 1 through 12), peri-pandemic period (discharges after March 8, 2020), and an interaction between peri-pandemic and rural. All models were weighted for pre/peri differences in demographic and clinical characteristics using entropy weights calculated separately for stays for each condition, aligning age, sex, and Elixhauser comorbidity index score for mortality. Standard errors were clustered on hospital ID.

eTable 5. Full Regression Models Comparing In-Hospital Mortality Among Non-Covid-19 Stays in 2020 and 2021, by Month, Relative to Prepandemic stays

Reason for hospital stay	2020									2021											
	Apr	May	Jun	Jul	Aug	Sep	Oct	Nov	Dec	Jan	Feb	Mar	Apr	May	Jun	Jul	Aug	Sep	Oct	Nov	Dec
**AMI (non-Covid)**																					
**Rural**																					
OR	**1.27**	1.10	1.11	0.96	1.12	1.04	1.08	1.08	1.02	**1.36**	**1.25**	1.12	**1.40**	1.02	1.00	1.02	**1.36**	**1.33**	1.12	1.21	0.81
Lower CI	**1.07**	0.93	0.93	0.79	0.94	0.87	0.92	0.90	0.85	**1.13**	**1.01**	0.91	**1.14**	0.81	0.79	0.77	**1.07**	**1.02**	0.83	0.83	0.60
Upper CI	**1.51**	1.31	1.32	1.17	1.33	1.24	1.26	1.31	1.23	**1.63**	**1.55**	1.38	**1.73**	1.28	1.26	1.35	**1.72**	**1.74**	1.51	1.76	1.10
**Urban**																					
OR	**1.16**	**1.17**	**1.13**	**1.15**	**1.17**	**1.12**	**1.12**	**1.18**	**1.10**	**1.18**	**1.11**	**1.10**	**1.14**	**1.18**	**1.10**	**1.12**	**1.21**	**1.31**	**1.23**	**1.30**	**1.25**
Lower CI	**1.09**	**1.10**	**1.07**	**1.09**	**1.11**	**1.06**	**1.06**	**1.11**	**1.04**	**1.10**	**1.04**	**1.03**	**1.06**	**1.10**	**1.02**	**1.02**	**1.10**	**1.19**	**1.07**	**1.12**	**1.08**
High CI	**1.23**	**1.24**	**1.20**	**1.22**	**1.24**	**1.19**	**1.18**	**1.24**	**1.16**	**1.27**	**1.20**	**1.17**	**1.22**	**1.26**	**1.19**	**1.24**	**1.33**	**1.45**	**1.42**	**1.50**	**1.45**
**Hip fracture (non-Covid)**																					
**Rural**																					
OR	1.25	**1.37**	1.11	1.04	1.14	1.20	**1.31**	**1.43**	1.01	1.04	1.13	0.71	0.92	**1.52**	0.74	0.97	1.27	1.11	0.98	1.40	0.85
Lower CI	0.95	**1.05**	0.83	0.78	0.85	0.90	**1.02**	**1.10**	0.75	0.72	0.79	0.49	0.61	**1.08**	0.48	0.61	0.80	0.71	0.54	0.85	0.45
Upper CI	1.64	**1.79**	1.48	1.39	1.52	1.60	**1.70**	**1.87**	1.36	1.50	1.61	1.05	1.39	**2.13**	1.12	1.54	2.03	1.74	1.78	2.31	1.58
**Urban**																					
OR	1.01	**1.16**	1.00	1.06	1.09	0.99	**1.15**	**1.16**	**1.15**	1.10	1.10	1.01	1.10	1.17	1.13	1.04	1.10	1.30	1.00	**1.54**	1.08
Lower CI	0.88	**1.02**	0.88	0.93	0.95	0.87	**1.01**	**1.03**	**1.01**	0.94	0.93	0.86	0.94	0.98	0.95	0.83	0.87	1.04	0.71	**1.17**	0.80
High CI	1.15	**1.31**	1.14	1.21	1.24	1.13	**1.30**	**1.32**	**1.30**	1.29	1.29	1.19	1.30	1.38	1.34	1.30	1.39	1.64	1.43	**2.03**	1.46
**GIH (non-Covid)**																					
**Rural**																					
OR	1.09	1.11	1.10	0.93	1.22	1.10	1.19	1.06	1.09	1.20	**1.44**	0.72	0.99	1.05	0.87	1.08	1.12	1.38	1.03	1.42	1.11
Lower CI	0.81	0.85	0.83	0.68	0.93	0.82	0.90	0.77	0.83	0.86	**1.05**	0.46	0.69	0.74	0.58	0.70	0.73	0.90	0.60	0.89	0.69
Upper CI	1.47	1.45	1.45	1.27	1.61	1.49	1.58	1.45	1.44	1.69	**1.98**	1.11	1.42	1.50	1.30	1.67	1.72	2.13	1.78	2.27	1.78
**Urban**																					
OR	**1.25**	1.06	1.07	**1.11**	**1.19**	1.09	0.99	**1.25**	1.08	**1.13**	1.02	0.94	1.11	1.03	1.11	1.15	**1.23**	**1.38**	1.05	1.20	0.95
Lower CI	**1.12**	0.96	0.96	**1.00**	**1.07**	0.98	0.89	**1.13**	0.98	**1.00**	0.89	0.83	0.97	0.90	0.97	0.94	**1.00**	**1.14**	0.79	0.93	0.74
High CI	**1.39**	1.18	1.18	**1.23**	**1.32**	1.22	1.10	**1.39**	1.20	**1.28**	1.16	1.07	1.27	1.17	1.27	1.40	**1.51**	**1.66**	1.40	1.56	1.21

© 2024 Jiang HJ et al. *JAMA Network Open*.

Reason for hospital stay	2020									2021											
	Apr	May	Jun	Jul	Aug	Sep	Oct	Nov	Dec	Jan	Feb	Mar	Apr	May	Jun	Jul	Aug	Sep	Oct	Nov	Dec
**Pneumonia (non-Covid)**																					
**Rural**																					
OR	**1.56**	**1.68**	**1.69**	**1.31**	**1.51**	**1.41**	**1.33**	**1.94**	**1.67**	**2.02**	**2.09**	**1.45**	**1.54**	**1.40**	**1.61**	**1.34**	**1.50**	**1.99**	**1.54**	**1.61**	**1.77**
Lower CI	**1.36**	**1.45**	**1.45**	**1.12**	**1.30**	**1.21**	**1.14**	**1.69**	**1.45**	**1.70**	**1.75**	**1.19**	**1.26**	**1.15**	**1.30**	**1.07**	**1.19**	**1.61**	**1.13**	**1.21**	**1.38**
Upper CI	**1.80**	**1.94**	**1.96**	**1.54**	**1.75**	**1.66**	**1.56**	**2.24**	**1.93**	**2.41**	**2.49**	**1.76**	**1.88**	**1.72**	**1.99**	**1.69**	**1.88**	**2.47**	**2.09**	**2.14**	**2.26**
**Urban**																					
OR	**1.70**	**1.58**	**1.40**	**1.25**	**1.28**	**1.37**	**1.27**	**1.44**	**1.64**	**1.83**	**1.77**	**1.74**	**1.51**	**1.55**	**1.41**	**1.29**	**1.46**	**1.72**	**1.55**	**1.57**	**1.64**
Lower CI	**1.57**	**1.46**	**1.28**	**1.14**	**1.17**	**1.26**	**1.16**	**1.32**	**1.51**	**1.65**	**1.59**	**1.57**	**1.34**	**1.38**	**1.25**	**1.11**	**1.24**	**1.47**	**1.23**	**1.28**	**1.37**
High CI	**1.85**	**1.72**	**1.53**	**1.36**	**1.40**	**1.50**	**1.38**	**1.57**	**1.78**	**2.03**	**1.96**	**1.93**	**1.70**	**1.75**	**1.59**	**1.51**	**1.71**	**2.00**	**1.95**	**1.92**	**1.96**
**Sepsis (non-Covid)**																					
**Rural**																					
OR	**1.43**	**1.35**	**1.28**	**1.25**	**1.43**	**1.31**	**1.32**	**1.51**	**1.54**	**1.64**	**1.49**	**1.31**	**1.35**	**1.36**	**1.33**	**1.32**	**1.60**	**1.82**	**1.87**	**1.64**	**1.66**
Lower CI	**1.34**	**1.26**	**1.19**	**1.17**	**1.34**	**1.22**	**1.23**	**1.40**	**1.43**	**1.51**	**1.36**	**1.21**	**1.24**	**1.24**	**1.21**	**1.17**	**1.43**	**1.62**	**1.60**	**1.43**	**1.44**
Upper CI	**1.52**	**1.44**	**1.37**	**1.35**	**1.52**	**1.40**	**1.42**	**1.62**	**1.66**	**1.79**	**1.62**	**1.42**	**1.47**	**1.50**	**1.46**	**1.48**	**1.79**	**2.04**	**2.18**	**1.88**	**1.91**
**Urban**																					
OR	**1.36**	**1.28**	**1.17**	**1.25**	**1.30**	**1.17**	**1.16**	**1.25**	**1.31**	**1.39**	**1.30**	**1.19**	**1.22**	**1.29**	**1.22**	**1.23**	**1.39**	**1.44**	**1.46**	**1.45**	**1.46**
Lower CI	**1.32**	**1.24**	**1.14**	**1.22**	**1.26**	**1.14**	**1.13**	**1.22**	**1.27**	**1.34**	**1.26**	**1.16**	**1.18**	**1.25**	**1.17**	**1.17**	**1.32**	**1.36**	**1.36**	**1.34**	**1.34**
High CI	**1.40**	**1.31**	**1.20**	**1.29**	**1.34**	**1.21**	**1.20**	**1.29**	**1.35**	**1.43**	**1.35**	**1.23**	**1.26**	**1.34**	**1.26**	**1.30**	**1.46**	**1.52**	**1.57**	**1.57**	**1.58**
**Stroke (non-Covid)**																					
**Rural**																					
OR	1.16	0.94	**1.30**	1.18	**1.26**	1.13	1.05	1.09	1.17	**1.34**	**1.43**	1.18	1.25	0.96	1.05	1.06	**1.65**	**1.39**	**1.66**	1.47	1.43
Lower CI	0.94	0.75	**1.07**	0.95	**1.03**	0.88	0.85	0.87	0.96	**1.04**	**1.13**	0.93	0.96	0.71	0.78	0.77	**1.22**	**1.01**	**1.06**	0.99	0.98
Upper CI	1.42	1.17	**1.58**	1.46	**1.55**	1.44	1.29	1.37	1.43	**1.73**	**1.80**	1.51	1.63	1.29	1.40	1.47	**2.23**	**1.90**	**2.61**	2.17	2.11
**Urban**																					
OR	1.03	1.00	1.01	1.02	1.05	0.97	0.95	0.97	1.00	**1.12**	1.09	1.02	0.98	**1.14**	1.09	1.10	**1.16**	1.03	1.13	**1.36**	1.14
Lower CI	0.96	0.93	0.94	0.95	0.98	0.90	0.88	0.91	0.93	**1.03**	1.00	0.93	0.90	**1.05**	1.00	0.96	**1.02**	0.91	0.95	**1.14**	0.93
High CI	1.11	1.07	1.08	1.09	1.13	1.04	1.02	1.04	1.07	**1.22**	1.18	1.12	1.07	**1.25**	1.19	1.26	**1.32**	1.18	1.35	**1.62**	1.40

Abbreviations: AMI, acute myocardial infarction; CI, confident interval; GIH, gastrointestinal hemorrhage; OR, odds ratio

© 2024 Jiang HJ et al. *JAMA Network Open*.

Note: Values in bold are statistically significant ($p<0.05$). Odds ratio estimates are from interrupted time-series logistic regression models. Separate models were estimated for each month and included the following independent variables: rural status (based on hospital location), peri-period (discharges after March 8, 2020), and an interaction between peri-pandemic and rural. All models were weighted for pre/peri differences in demographic and clinical characteristics using entropy weights calculated separately for stays for each condition, aligning age, sex, and Elixhauser comorbidity index score for mortality. Standard errors were clustered on hospital ID.

© 2024 Jiang HJ et al. *JAMA Network Open*.

eFigure 1. Odds of In-Hospital Mortality Among Non-Covid-19 Stays for GI Hemorrhage, Hip Fracture, and Stroke During March 8–December 31, 2020, Overall and by Covid-19 Burden in the Hospital's Community, Relative to Prepandemic Stays**.**

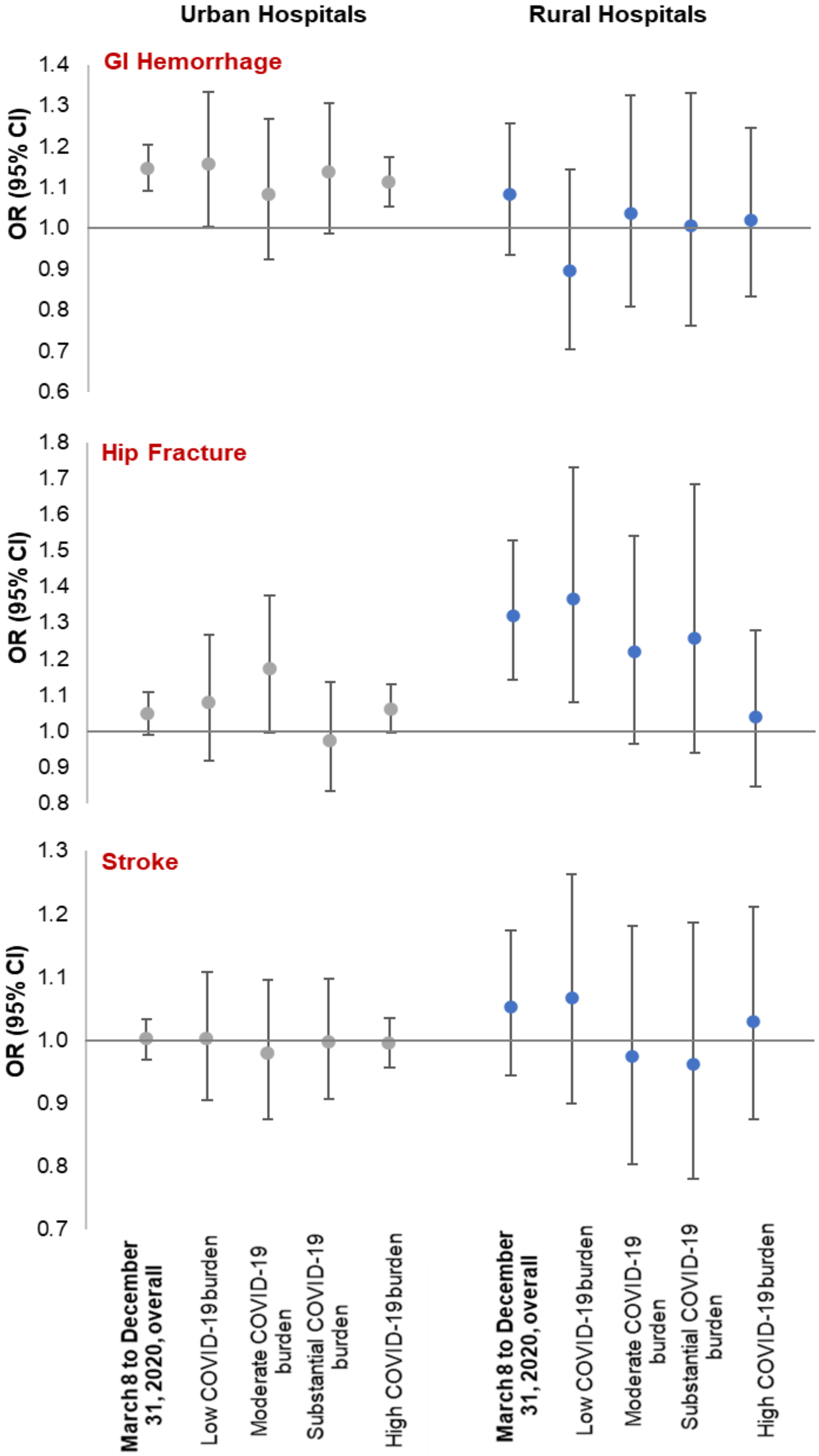

Notes: CI, confidence interval; OR, odds ratio. Because not all states had data in 2021, these results are limited to the 2020 peri-pandemic period (March 8–December 31, 2020).

© 2024 Jiang HJ et al. *JAMA Network Open*.

eFigure 2. Odds of In-Hospital Mortality Among Non-Covid-19 Stays for GI Hemorrhage, Hip Fracture, and Stroke in 2020 and 2021, by Month, Relative to Prepandemic Stays

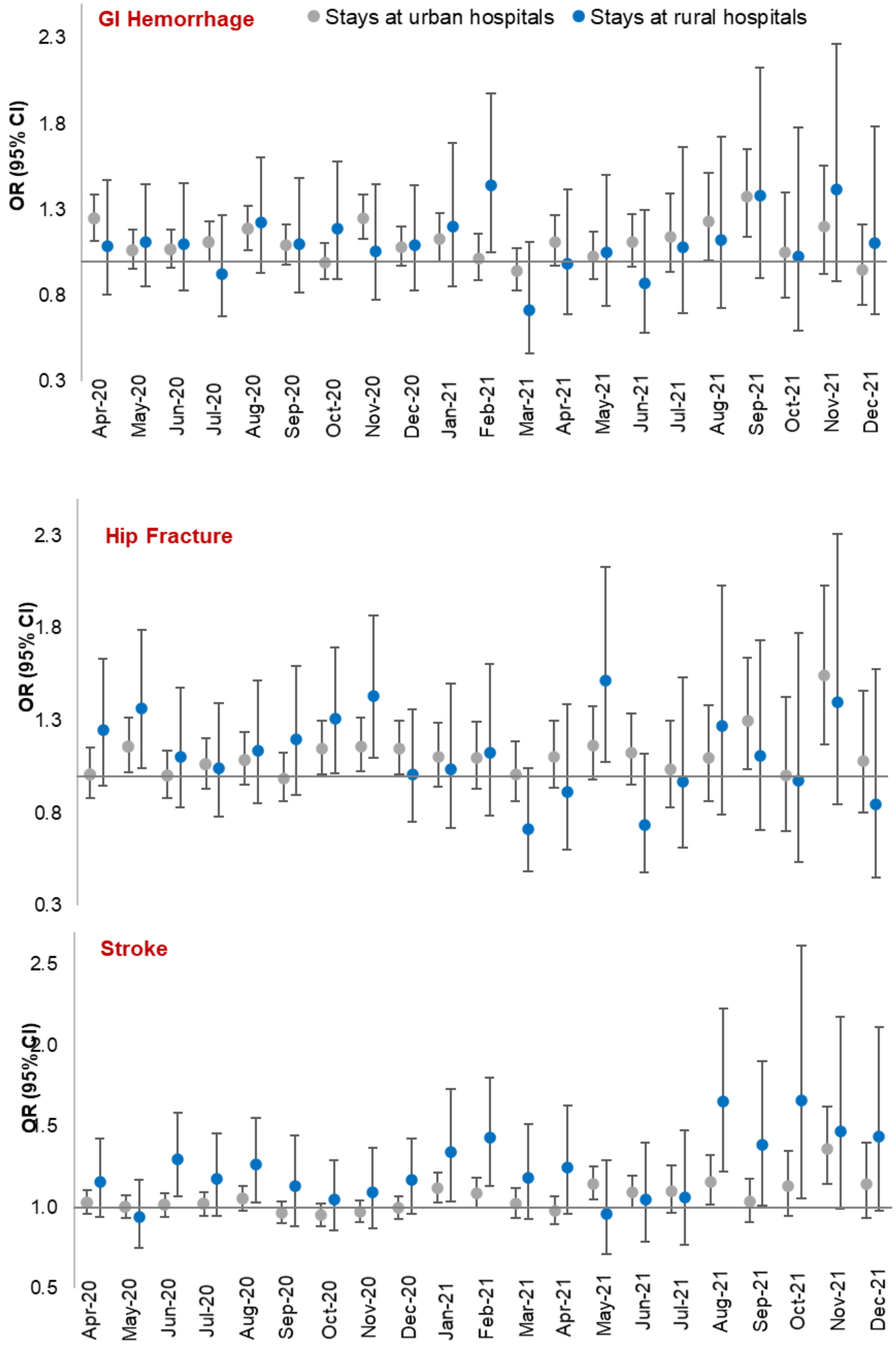

Note: CI, confidence interval; OR, odds ratio.

© 2024 Jiang HJ et al. *JAMA Network Open*.

eFigure 3. Percentage of Hospitals in Counties with High Community COVID-19 Burden (100+ Cases per 100,000 Population in the Past 7 Days), 2020-2021

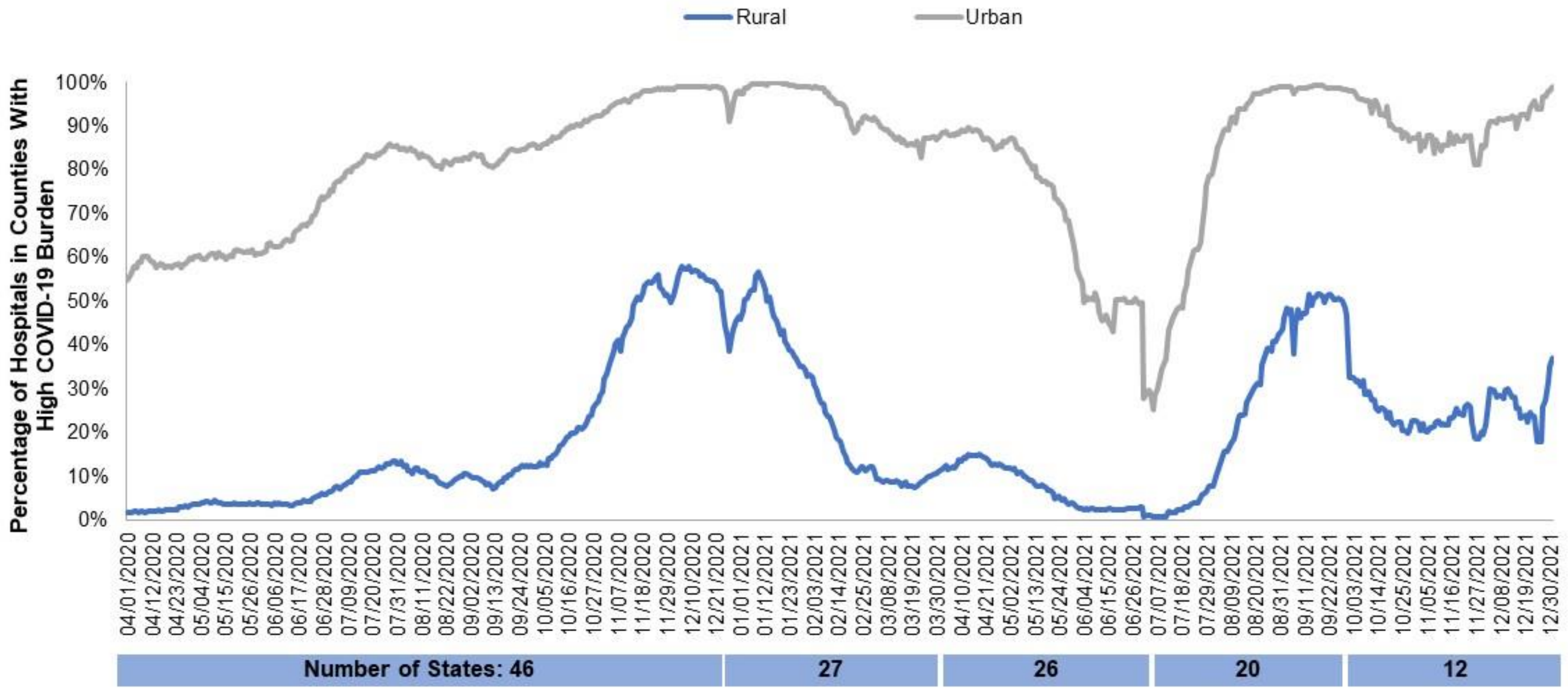

SOURCES Agency for Healthcare Research and Quality (AHRQ), Healthcare Cost and Utilization Project (HCUP), State Inpatient Databases (SID), 2017-2021 and USA Facts, 2020-2021

© 2024 Jiang HJ et al. *JAMA Network Open*.

eReferences

1. Clinical Classifications Software Refined (CCSR) for ICD-10-CM Diagnoses. Healthcare Cost and Utilization Project (HCUP). Rockville, Md.: Agency for Healthcare Research and Quality, February 2022. (https://www.hcup-us.ahrq.gov/toolssoftware/ccsr/dxccsr.jsp.)
2. Federal Office of Rural Health Policy (FORHP) Data Files. Rockville, Md.: Health Resources and Services Administration, March 2022. (https://www.hrsa.gov/rural-health/about-us/what-is-rural/data-files.)
3. Hainmueller J, Xu Y. ebalance: a Stata package for entropy balancing. J Stat Softw 2013;54:1-18.
4. Elixhauser Comorbidity Software Refined for ICD-10-CM. Healthcare Cost and Utilization Project (HCUP). Rockville, Md.: Agency for Healthcare Research and Quality, October 2021. (www.hcup-us.ahrq.gov/toolssoftware/comorbidityicd10/comorbidity_icd10.jsp.)
