# Supplementary material for: Mortality for Time-Sensitive Conditions at Urban vs Rural Hospitals During the COVID-19 Pandemic
Source: JAMA Netw Open. 2024 Mar 12;7(3):e241838. doi: 10.1001/jamanetworkopen.2024.1838 (PMC10933716; doi:10.1001/jamanetworkopen.2024.1838)
Supplement: Supplement 2. — Data Sharing Statement [file jamanetwopen-e241838-s002.pdf]

Data Sharing Statement

Jiang. Mortality for Time-Sensitive Conditions at Urban vs Rural Hospitals During the COVID-19 Pandemic. *JAMA Netw Open*. Published March 12, 2024. doi:10.1001/jamanetworkopen.2024.1838

Data
Data available: No
